# Mechanism of Rad26-assisted rescue of stalled RNA polymerase II in transcription-coupled repair

**DOI:** 10.1038/s41467-021-27295-4

**Published:** 2021-12-01

**Authors:** Chunli Yan, Thomas Dodd, Jina Yu, Bernice Leung, Jun Xu, Juntaek Oh, Dong Wang, Ivaylo Ivanov

**Affiliations:** 1 Department of Chemistry, Georgia State University, Atlanta, GA USA; 2 Center for Diagnostics and Therapeutics, Georgia State University, Atlanta, GA USA; 3 Division of Pharmaceutical Sciences, Skaggs School of Pharmacy & Pharmaceutical Sciences, University of California San Diego, La Jolla, CA 92093 USA; 4 Department of Cellular & Molecular Medicine, School of Medicine, University of California San Diego, La Jolla, CA 92093 USA; 5 Department of Chemistry and Biochemistry, University of California San Diego, La Jolla, CA 92093 USA

**Keywords:** Molecular modelling, Computational biophysics, Nucleotide excision repair

## Abstract

Transcription-coupled repair is essential for the removal of DNA lesions from the transcribed genome. The pathway is initiated by CSB protein binding to stalled RNA polymerase II. Mutations impairing CSB function cause severe genetic disease. Yet, the ATP-dependent mechanism by which CSB powers RNA polymerase to bypass certain lesions while triggering excision of others is incompletely understood. Here we build structural models of RNA polymerase II bound to the yeast CSB ortholog Rad26 in nucleotide-free and bound states. This enables simulations and graph-theoretical analyses to define partitioning of this complex into dynamic communities and delineate how its structural elements function together to remodel DNA. We identify an allosteric pathway coupling motions of the Rad26 ATPase modules to changes in RNA polymerase and DNA to unveil a structural mechanism for CSB-assisted progression past less bulky lesions. Our models allow functional interpretation of the effects of Cockayne syndrome disease mutations.

## Abstract (web summary)

Here the authors provide models of RNA polymerase II bound to the yeast CSB ortholog Rad26 in different nucleotide states; explain how Rad26 domain motions help the polymerase progress past DNA lesions; and interpret the effects of CSB-associated disease mutations.

## Introduction

Genomic DNA is under constant assault from a host of endogenous and environmental factors causing DNA damage. Nucleotide excision repair (NER) is arguably the most versatile pathway to repair damaged DNA. NER has evolved to remove a wide array of DNA lesions from the genome, including cyclobutane–pyrimidine dimers (CPD) and 6–4 pyrimidine–pyrimidone (6–4 PP) photoproducts, which are the major lesions induced by ultraviolet radiation; intrastrand crosslinks caused by cancer therapeutic drugs such as cisplatin; cyclopurines generated by reactive oxygen species; and bulky chemical adducts caused by carcinogen exposure^1–3^. NER is also exceptional for the variety of clinical manifestations associated with its genetic impairment^1,4–6^. Deficiencies in NER’s two sub-pathways, transcription-coupled repair and global genome repair, cause severe human diseases^7–10^—ultraviolet radiation‑sensitive syndrome (UVSS), the genetic disorder xeroderma pigmentosum (XP) linked to extreme cancer predisposition, cerebro-oculo-facio-skeletal syndrome (COFS), trichothiodystrophy (TTD), and Cockayne syndrome (CS) spectrum of disorders associated with premature ageing and accelerated neurodegeneration. This striking clinical heterogeneity is still incompletely understood at the level of structure and biological mechanisms.

In particular, transcription-coupled repair^11–13^ is essential for lesion removal from the template strand during transcription and is activated by the recruitment of Cockayne Syndrome B protein (CSB/ERCC6) to lesion-arrested RNA polymerase II (Pol II)^14–19^. In turn, CSB association triggers recruitment of downstream NER factors, such as Cockayne syndrome protein A (CSA/ERCC8), transcription factor IIH (TFIIH), and UV-sensitive syndrome protein (UVSSA)^20,21^. Mutations in CSB are associated with Cockayne syndrome, an autosomal-recessive genetic disorder, characterized by postnatal growth failure, progressive neurological dysfunction, premature aging, and photosensitivity^22,23^. Among Cockayne syndrome patients there is a high incidence of mutations in the CSB gene (~70%) with over 80 disease mutations characterized to date^24,25^.

**Fig. 1:**
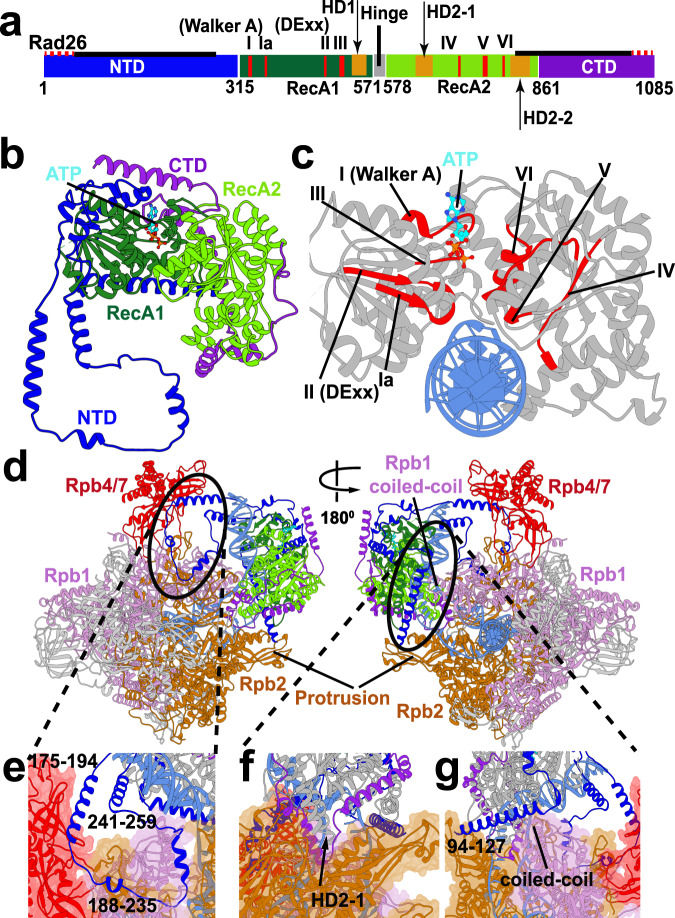
Cryo-EM based Pol II–Rad26 structural model reveals the critical anchoring roles of the NTD and CTD domains. **a** Schematic showing the Rad26 protein domains along its sequence. Newly modeled regions are highlighted with solid black lines. Seven canonical SF2 helicase motifs are shown in red bars. The hinge between the two ATPase modules is indicated by a black vertical bar. **b** View of Rad26 in cartoon representation colored by domains. RecA1 is shown in green, RecA2 in light green, NTD in blue and CTD in purple. **c** Side view of the Rad26 active site cleft with DNA (blue) and highlighted functional motifs I–VI (red). ATP is shown in stick representation and colored in cyan. **d** Anterior and posterior views of the Pol II–Rad26 complex. Rpb1, Rpb2 and Rpb4/7 are in light purple, brown and red, respectively. Rad26 is colored by domains. Circles demark zoomed-in regions in panels e-g. **e** NTD of Rad26 interacting with the Rpb1, Rpb2 and Rpb4/7 subunits of Pol II. **f** CTD and HD2-1 of Rad26 interacting with Rpb2 and DNA. **g** NTD of Rad26 interacting with the Rpb1 protrusion and the clamp coiled-coil domain.

Despite decades of genetic and biochemical studies, the role of CSB-Pol II association in the early stages of transcription-coupled repair remains incompletely understood^11,26^. Insights have come from analysis of yeast CSB orthologs, Rad26 in *Saccharomyces cerevisiae* and Rhp26 in *Schizosaccharomyces pombe*^2,27–30^. Notably, the recent cryo-EM structure of a Pol II–Rad26 complex was a breakthrough, revealing the binding mode of Rad26 and the prominent change in the DNA path induced by its presence^28^. Rad26 belongs to the SWI2/SNF2 family of chromatin remodelers. SWI2/SNF2 proteins share a common catalytic core, comprised of two RecA-like domains (RecA1, RecA2), which by themselves are sufficient to power DNA remodeling. In Rad26, RecA1 and RecA2 are separated by a flexible hinge region (residues 571-578) to facilitate the relative motion of the two domains during each cycle of ATP hydrolysis and DNA translocation (Fig. 1)^28,30^. The domains feature a set of seven conserved ATPase sequence motifs (Fig. 1a, c; Supplementary Fig. 1). The RecA1 motifs (I, Ia, II, and III) are primarily responsible for binding ATP, while the RecA2 motifs (IV, V and VI) mediate upstream dsDNA binding and, thus, may have a role in energy transduction. Three additional motifs (Supplementary Fig. 1, 2a) have putative roles in dsDNA stimulation of Rad26 ATPase activity (IIa) or ATP-driven translocation (IVa and Va)^31^. Importantly, the Rad26 ATPase modules have pronounced structural similarity to DExx box helicases, suggesting a shared ATP hydrolysis mechanism and similar nucleotide-induced conformational changes^32^. Moreover, there is evidence from biochemistry that Rad26 can function as a molecular motor to promote the forward movement of Pol II along the DNA template^28,30^. However, no CSB/Rad26 structure has been captured with bound nucleotides, leaving an important gap in our understanding of the ATP hydrolysis cycle of Rad26 and its role in remodeling the transcription bubble of a stalled RNA polymerase.

To address this gap, we extended the structural model of the nucleotide-free Pol II–Rad26 complex to include previously unmodeled regions. We also created structural models of Pol II–Rad26 in the nucleotide-bound ATP and ADP states. We then employed extensive molecular dynamics simulations and applied novel graph-theoretical approaches^33^ to define dynamic networks, communities and allosteric communication mechanisms in all three functional states of the Pol II–Rad26 assembly. Our analyses uncovered the structural elements, key protein residues and allosteric paths coupling ATP-hydrolysis to DNA binding and unveiled a possible structural basis for Rad26-mediated DNA remodeling to assist Pol II progression past damaged sites on DNA. Our computational analyses were complemented with mutational experiments, which validated the significance of key residues in the Pol II–Rad26 dynamic network.

## Results

### Reconstruction of a complete Pol II–Rad26 complex including the N- and C-terminal domains of Rad26

The previous Pol II–Rad26 cryo-EM structure was significant for offering an unprecedented molecular view of this complex protein machinery and explaining the functional role of Rad26 in early transcription-coupled repair^28^. The structure revealed how Rad26 engages the Pol II elongation complex, binding the upstream DNA duplex and dramatically altering its path. Importantly, it supported a mechanism wherein Rad26 recognizes stalled Pol II and acts as a molecular motor to reduce Pol II backtracking, promote forward translocation on the DNA template and facilitate transcriptional bypass of less bulky DNA lesions^28^. While Pol II and the Rad26 ATPase core could be modeled with confidence into the cryo-EM density at 5.8 Å resolution, the N- and C-terminal domains (NTD and CTD) remained unmodeled. Previous studies revealed that the NTD and CTD domains of *S. cerevisiae* Rad26, its *S. pombe* ortholog Rhp26 and human CSB play critical roles in regulating the ATPase and chromatin remodeling/DNA translocase activities of the central core domain and are involved in the recruitment of downstream repair factors and in UV damage dependent chromatin association^20,34–38^. Therefore, inclusion of these regions is essential for the success of molecular simulations aimed at elucidating the functional dynamics of the Pol II–Rad26 assembly.

To shed light on the functional roles of the CTD and NTD, we built a complete structural model of the Pol II–Rad26 complex from the available cryo-EM densities (EMD-8735 and EMD-8736)^28^. We used structures of SWI2/SNF2 proteins (PDB ID: 5HZR^39^, 5X0Y^40^, and 5JXR^41^) as templates for homology modeling to reconstruct the missing residues from the C-terminal end of Rad26 (798–861), which were subsequently docked into the cryo-EM density (EMD-8735). The missing residues from the NTD (94–228) and CTD (862–1085) were traced in the original EM density and built de novo. Rad26 and Pol II were then separately flexibly fitted into the cryo-EM density (EMD-8735) and combined to assemble the final model. The newly modeled regions of Rad26 are shown in Fig. 1 and Supplementary Fig. 3.

The Rad26 N-terminal domain is comprised of five extended helices connected by long linkers. As a result, the NTD is very extended and traverses the Pol II surface, making extensive contacts with Rpb1, Rpb2 and Rpb4/7 while also wrapping around the RecA1 domain (Fig. 1b, Supplementary Fig. 3a, and Supplementary Movie 1). Notably, a segment of NTD residues (171–264) lodges between the Pol II stalk (Rpb4/Rpb7) and the Rad26 RecA1 domain with linker residues (188–235) tracking along the Pol II Rpb1 and Rpb2 subunits. Importantly, along the path of the NTD are functionally significant elements of Pol II (Fig. 1e, g, Supplementary Fig. 3c, e, and Supplementary Movie 1), e.g. the Rpb1 coiled-coil domain and the Pol II stalk, which are common docking sites for transcription factors. Additionally, two helices from the Rad26 NTD domain make direct contacts with the upstream DNA duplex and the upstream fork of the DNA bubble (Fig. 1e, g, Supplementary Fig. 3c, e, and Supplementary Movie 1). Collectively, these contacts provide a handle for Rad26 to 1) exert regulatory influence on Pol II by affecting the binding of transcription factors; and 2) affect the stability of the transcription bubble and Pol II translocation along DNA. Tethering of Rad26 to Pol II via the NTD and the positioning of NTD between the Rad26 ATPase core and the RNA polymerase are key for maintaining the structural integrity of the entire Pol II–Rad26 assembly. Consistent with these observations, Rad26 becomes entirely or partially dispensable for transcription-coupled repair in yeast cells in the absence of Rpb4^29,42^.

The C-terminal domain of Rad26 is also predominantly helical and wraps around the back side of the ATPase core, serving as a latch between the RecA1 and RecA2 domains (Fig. 1b, Supplementary Fig. 3a, and Supplementary Movie 1). Notably, residues from the CTD (883–919) and the HD2-1 linker (593–632) intercalate between the two DNA strands at the fork of the upstream transcription bubble (Fig. 1f, Supplementary Fig. 2b and 3d**;** Supplementary Movie 1). Indeed, we previously identified a CSB-specific coupling motif in the CTD (residues 900–910 in Rhp26 and 904–914 in Rad26) that couples the ATPase and chromatin remodeling/translocation activities^37^. The interactions between the newly modeled CTD and HD2-1 linker may explain how this region couples the ATPase and chromatin remodeling/translocation activities. The NTD and CTD of Rad26 pack against the HD1 and HD2 insertions of the ATPase domain to form key interactions with the upstream DNA fork, which is crucial for stabilizing the DNA bubble (Supplementary Fig. 2b). Moreover, in order to function as a molecular motor on dsDNA, Rad26 requires stable attachment to Pol II. In this respect, the newly modeled CTD and the NTD regions are critical. Together, the CTD and NTD contribute 1649 Å^2^ (71%) of buried surface area to the overall Rad26–Pol II interface, making them indispensable for the structural integrity of the complex (Supplementary Table 1).

### Structural basis for Rad26 conformational switching during the ATPase cycle

**Fig. 2:**
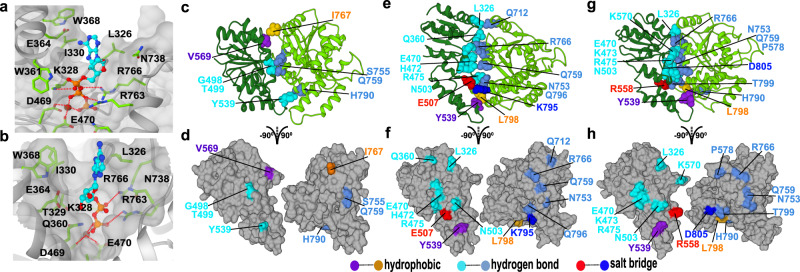
Structural models elucidate the conformational switching of Rad26 in response to nucleotide binding and its crucial role in transcription bubble remodeling. **a** View of the Rad26 active site with bound ATP. **b** View of the Rad26 active site with bound ADP. The binding pocket is shown in gray. Nucleotides are shown in ball-and-stick representation (cyan). Select residues making key contacts with the nucleotide are shown in stick representation (green). Red dash lines denote hydrogen bonds. Mg (dark brown sphere) is coordinated by D469, D470 and ATP/ADP phosphate (red dash lines). (**c**, **e**, **g**) Residue contacts at the RecA1 and RecA2 interface in (**c**), apo state; (**e**), ATP state; (**g**), ADP state. (**d**, **f**, **h**) Residue contacts mapped onto the RecA1 and RecA2 surface in (**d**). apo state; (**f**), ATP state; (**h**), ADP state. The side chains of contact forming residues are shown with salt–bridge interactions in red and blue, hydrogen bonding interactions between polar residues in cyan and light blue, and hydrophobic interactions in orange and purple.

To shed light on the nucleotide-induced conformational changes during the Rad26 ATP hydrolysis cycle, we modeled the ATP- and ADP-bound states of the Pol II–Rad26 complex based on a yeast SNF2 structure obtained with ADP-BeFx as a ligand^43^. We then simulated the Pol II–Rad26 assembly for ~ 11 μs in each of three functional states—apo, ATP and ADP. Clustering analysis of the MD trajectories past the point of convergence allowed us to identify the dominant conformers (Fig. 2) and uncover the intricate conformational rearrangements of the Rad26 ATPase core during the ATP-hydrolysis cycle. The observed binding modes of the ATP and ADP nucleotides are shown in Fig. 2a, b, respectively. Nucleotide binding occurs at the interdomain cleft formed by RecA1 and RecA2. Residues from the conserved ATPase motifs form the primary nucleotide-binding pocket: L326, K328, I330 from motif I, D469 and E470 from motif II, and R763 and R766 from motif VI. Specifically, the adenine group is wedged between the hydrophobic L326, I330, and W368 residues. In addition, five residues associated with motifs I, II, and VI (K328, R763, R766, D469, and E470) contact the phosphate moiety either directly or indirectly through Mg^2+^ ion coordination. Based on the Sth1 and Snf2 structures^43,44^, the residues functionally equivalent to R763 and R766 are coordinated and positioned to serve as sensors of the nucleotide state of the active site. Additionally, there is mutational evidence of the functional significance of the sensor residues^31,45^. Notably, even conservative mutation of K328 to arginine completely abolishes Rad26 ATPase activity^28^. Similarly, in CSB mutation of E646, which is the functional equivalent of E470 in Rad26, abolishes ATPase activity whereas mutations in motifs V and VI diminish the ATP-hydrolysis activity^46^.

Nucleotides induce mutual rotation of the RecA1 and RecA2 domains accompanied by closure of the interdomain cleft (Supplementary Movie 2). Correspondingly, the RecA1–RecA2 interface becomes progressively tighter from the apo state to the ADP-bound and eventually to the ATP-bound conformer (Fig. 2c–h). These changes are reflected in the dramatic increase in buried surface area (BSA) at the domain interface. The loosely assembled apo interface features BSA of only 866 Å^2^ while the ADP-bound (1293 Å^2^) and ATP-bound (1322 Å^2^) interfaces are considerably more extended. The same progression is evident in the computed B-factors for the Rad26 core (Supplementary Fig. 4a–l), showing decreased mobility and increased interface stability going from the apo to the ADP and the ATP functional states.

To gain insight into the residue interactions responsible for the observed changes, we relied on persistent contact analysis of the MD trajectories. Specifically, we classified interface contacts as hydrogen-bond, salt–bridge or hydrophobic and defined suitable geometric criteria for each class of interactions to identify them in the simulation trajectories. Contacts occurring in more than 50% of the MD trajectory frames were considered persistent. Predictably, the RecA1–RecA2 interface is dominated by persistent contacts between conserved polar and charged residues (Fig. 2c–h and Supplementary Fig. 4g–i). Electrostatics plays a critical role in accommodating the negative charge of ATP by positioning the ligand between two overwhelmingly positive Rad26 surfaces (Supplementary Fig. 4g–i). This also ensures a marked decrease in interface compactness upon release of the negative γ-phosphate after ATP hydrolysis. Correspondingly, in the apo state RecA1 is rotated away from RecA2 leaving a significant gap between the ATPase modules. The domain interface is held together by a cluster of hydrogen bonding residues (G498, T499, Y539 from RecA1 and S755, Q759, H790 from RecA2) and a few additional contacts e.g. the hydrophobic V569-I767 pair (Fig. 2c, d). By contrast, the presence of ATP reorients the ATPase modules resulting in a wide, compact, electrostatically compatible interface between RecA1 and RecA2. The interface features numerous persistent contacts between conserved Rad26 functional motifs—I, II, IIa and HD1 in RecA1 and Va, VI and HD2 in RecA2 (Fig. 2 and Supplementary Fig. 1). Hydrogen bonding (Q360-R766_,_ K570-P578, L326-Q726), salt–bridge (E507-K795, R558-D805, K328-ATP, R766-ATP, R763-ATP) and hydrophobic interactions (W368-ATP, L326-ATP, I330-ATP, Y539-L798) are all present, strengthening RecA1-RecA2 domain association.

### Motions of the Rad26 ATPase domains upon change of nucleotide state result in direct DNA pulling

**Fig. 3:**
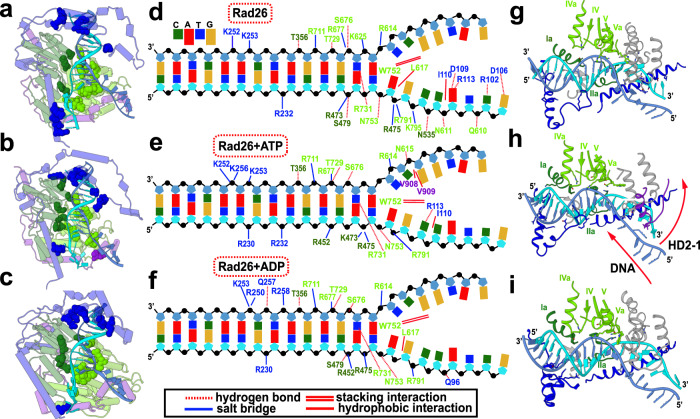
Nucleotide binding alters Rad26–DNA interactions. (**a**–**c**) Rad26 with highlighted Rad26–DNA contacts in (**a**), apo state; (**b**), ATP state; (**c**), ADP state. Rad26 is shown in cylindrical representation and colored by domains. The side chains of residues participating in DNA binding are shown as spheres and colored by domain—NTD in blue, RecA1 in dark green, RecA2 in light green and CTD in purple. (**d**–**f**), Schematic detailing the persistent contacts between Rad26 and DNA in (**d**), apo state; (**e**), ATP state; (**f**), ADP state. Residue labels are colored by domain. DNA phosphate groups are shown as black spheres. (**g**–**i**) Primary Rad26–DNA interactions in (**g**), apo state; (**h**), ATP state; (**i**), ADP state. Location of ATPase motifs on RecA1 and RecA2 are shown in dark green and light green, respectively.

Our Pol II–Rad26 models also shed light on the key question of how Rad26 harnesses the energy from ATP hydrolysis to effect conformational changes in DNA. Rad26 features a DNA binding groove formed by the RecA1 and RecA2 domains (Fig. 3a, d, g). DNA recognition involves charged contacts along the minor groove of the DNA duplex immediately preceding the transcription bubble. These interactions involve structural elements from the conserved Rad26 motifs (Ia and IIa from RecA1; IV, IVa, V, and Va from RecA2; Fig. 3g–i). Important contacts are also made by the RecA2 domain with single-stranded DNA at the fork of the transcription bubble. Among the RecA2 contacts, a tryptophan residue, W752 from motif Va, stands out. W752 is conserved not only in the CSB protein subfamily, but also in the Chd1, Snf2, ISWI, and INO80 chromatin remodelers (Supplementary Fig. 5). The bulky tryptophan side chain inserts into the upstream DNA fork and is accommodated through base-stacking interactions (Fig. 3 and Supplementary Fig. 5), highlighting the importance of W752 for stabilizing the transcription bubble and for DNA translocation. W752 is also adjacent to the N-terminus of motif VI, which harbors the conserved arginine residues (R763 and R766) essential for ATP hydrolysis. Therefore, W752 may provide a dual connection, on one side to the Rad26 ATP sensing elements, and on the other to the DNA fork. Strikingly, mutation of the functionally equivalent W936 residue in CSB has been reported to cause type I Cockayne syndrome^47^. Another key observation is that the number of persistent DNA contacts is much greater for the RecA2 domain as compared to RecA1. Weaker DNA binding allows RecA1 to slide along the minor groove during the translocation step while the RecA2 maintains a tighter grip at the fork of the transcription bubble (Fig. 3 and Supplementary Movie 2).

Importantly, our Pol II–Rad26 models display prominent differences in the DNA binding modes of the apo and nucleotide-bound conformers. The conformational transition from the compact ATP-bound state to the more open ADP-bound and apo states is accompanied by concerted swing motions of the RecA1 and RecA2 modules on the opposing sides of the upstream DNA duplex. In response, the duplex shifts back and rotates by 14° in the Rad26 DNA-binding groove (Supplementary Movie 2). Thus, DNA translocation induced by the Rad26 molecular motor occurs by stepwise winding and pushing along the axis of the upstream DNA duplex. The basic features of this translocation mechanism are shared with the bacterial Mfd protein, which was recently visualized by cryo-EM^48^. Strikingly, Rad26 and Mfd appear to have achieved functional convergence despite having no sequence conservation and only limited structural similarity.

In the apo state, structural elements from the HD1, HD2-1 and HD2-2 motifs and the CTD contact the ssDNA of the template strand via hydrogen-bond, salt–bridge and hydrophobic interactions. This tight grip on the ssDNA in the apo state allows RecA2 to act as a ratchet to prevent DNA slippage during the ATP-hydrolysis cycle. In contrast, weakened RecA2–ssDNA interactions in the nucleotide-bound states could allow easy DNA translocation. Switching of nucleotide state thus results in alternating weak and strong DNA binding at the edge of the transcription bubble and in Rad26-mediated pulling on the template strand. Collectively, these motions may facilitate lesion scanning in early transcription-coupled repair.

### Network analysis unveils Pol II–Rad26 dynamic communities and key allosteric communication mechanisms

To discover Pol II–Rad26 allosteric residue networks and communication mechanisms, we applied graph-theoretical approaches^33,49,50^ that map dynamic information from our extensive MD simulations onto graphs representing the protein topology (i.e. residues are represented by graph nodes; edges connect contacting residues). Graph edges are weighted by persistent contact probabilities derived from our MD ensembles, allowing allosteric communication to be quantified by the graph edge betweenness measure. We then applied the Girvan-Newman algorithm to partition the complexes into dynamic communities—tightly connected clusters of residues that move together as modules.

To accomplish systematic comparisons of allosteric responses in all three simulated complexes, we used the difference contacts network analysis method (dCNA)^51^. Briefly, dCNA involves the following steps: (1) individual residue contact networks are computed for each simulated complex separately; (2) a consensus network is calculated, in which edges represent stable contacts across all simulations; (3) communities are detected and mapped onto the consensus network; 4) subtracting contact probabilities of the individual networks leads to difference contact networks represented as graphs, indicating which communities/interfaces are gaining or losing stable contacts. In this way, dCNA maps multiple MD ensembles onto a single consensus network graph in order to monitor contact probability changes across community interfaces. In turn, this mapping reveals intricate contact differences and subunit interface rearrangements, indicative of allosteric communication.

**Fig. 4:**
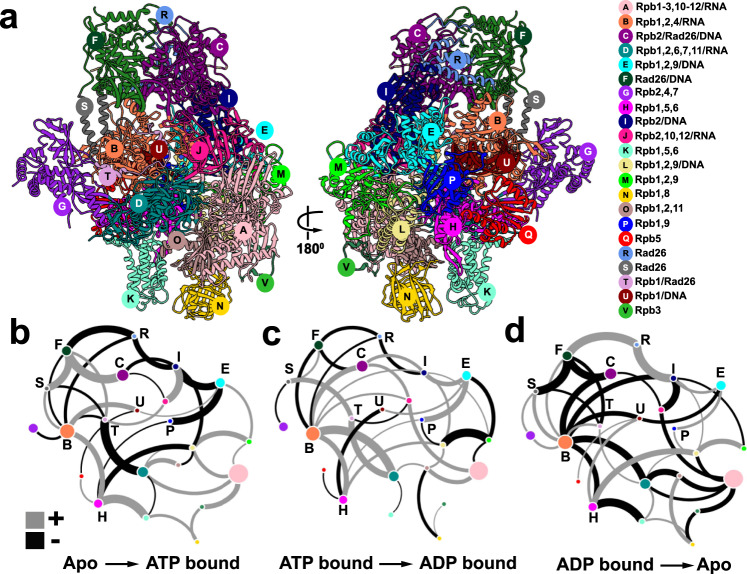
Community networks underlying Pol II–Rad26 functional dynamics during the ATPase cycle. **a** Consensus communities are identified from dynamic network analysis and mapped to the structure of Pol II–Rad26 complex. Communities are color-coded and labeled. Net change in contact probabilities across Pol II–Rad26 communities during the ATP hydrolysis cycle: (**b**), apo to ATP transition; (**c**) ATP to ADP transition; (**d**) ADP to apo transition. The radius of community vertex is correlated to the number of residues included in the community. The gray and black edges between communities (betweenness) indicate gain(+)/loss(−) of dynamic contacts.

**Fig. 5:**
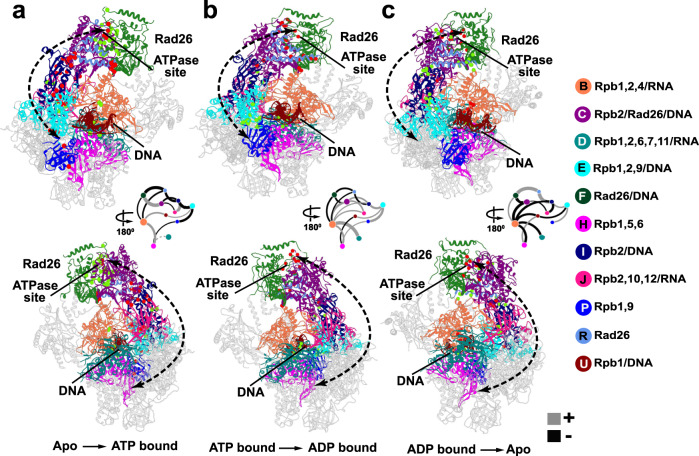
Discovery of an extended allosteric communication path through the Pol II–Rad26 complex from dynamic network analysis. Dynamic communities directly traversed by the allosteric path (black dotted arrow) are shown in color. dCNA subgraphs for these communities of the Pol II–Rad26 complex are shown as insets, indicating gain(+)/loss(−) of dynamic contacts for the following transitions: (**a**) apo to ATP; (**b**) ATP to ADP; (**c**) ADP to apo. Non-participating communities are shown in gray. Red and green spheres denote residues with highest gain/loss of contact probability mapped onto the Pol II–Rad26 structures.

We constructed a consensus network from ensemble simulations of the three Pol II–Rad26 functional states and identified 22 distinct communities (Fig. 4a, Supplementary Movie 3). Changes in contact probabilities between communities during the ATP hydrolysis cycle (apo → ATP-bound → ADP-bound → apo) are shown in Fig. 4b–d. Residues responsible for the largest changes in contact probability are mapped back onto the Pol II–Rad26 structure and shown in Fig. 5a–c. Rad26 encompasses five distinct communities. RecA1 and RecA2 correspond to communities F and C (Fig. 4a–d), which separate precisely along the ATP-binding cleft reflective of their function as independent ATPase modules. Community R covers the CTD latch at the back of the Rad26 ATPase core while communities S and T represent segments of the extended N-terminal domain. Notably, communities subdivide the complex into dynamic modules independently from the Pol II–Rad26 domain structure. For instance, community C includes not only RecA2 but also tightly associated parts of Rpb2 and DNA. Predictably, upon ATP binding we observe a large contact probability increase between communities C and F. This gain of contacts at the RecA1–RecA2 interface does not occur in isolation but, instead, triggers concomitant net contact losses between several adjacent communities (e.g. between the F–R, R–I, C–I, I–E community pairs). Strikingly, this change in interfacial contacts is not confined to Rad26 but extends from the Rad26 docking site on Rpb2 (community I) through the Rpb2 lobe (community E) all the way to the Rpb1 jaw (community P). Conversely, on the opposite side of Pol II the Rpb1 clamp (community B) and cleft (community H) gain stable contacts with one another and with the downstream dsDNA (community U). Together, these changes indicate a cascading allosteric response along the Pol II central cavity, which surrounds the transcription bubble. As expected for a cyclic process, the observed changes are partially reversed in the ATP to ADP transition and then fully reversed in the ADP to apo transition. Correspondingly, two-stage loss of contact probability is seen between communities C and F as the RecA1–RecA2 interface opens up on the way back to the apo state. On the other hand, interactions between RecA2 and the Rpb2 are enhanced in the ATP$\to$ADP transition suggesting the ATP-hydrolysis affects the RecA2 binding to Rpb2 and the DNA fork (community I). In the ADP$\to$apo transition we observe net contact gain across the interfaces of communities R, I and E via the same allosteric network.

Notably, dCNA detects a global allosteric network encompassing not only CSB but extending along the circumference of the Pol II central cavity. Our results support a mechanism whereby allosteric changes initiated within the Rad26/CSB molecular motor propagate to Pol II and, in turn, affect the stability of the transcription bubble and the opening/closing dynamics of the Pol II cleft. Such changes may allow Pol II to bypass lesions in the transcribed strand.

### Models shed light on the impact of CS disease mutations on CSB structure and dynamics

**Fig. 6:**
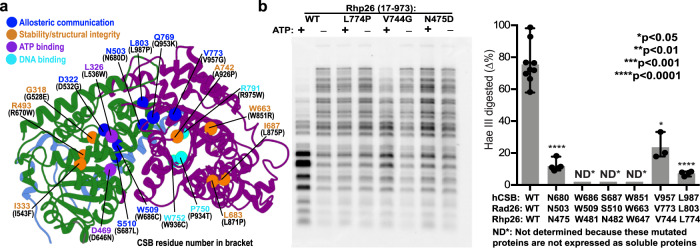
Cockayne syndrome disease mutations occupy distinct positions in the Rad26 dynamic network. **a** Cockayne syndrome missense disease mutations (sphere) mapped onto Rad26 and classified by type of structural or dynamic disruption they induce. Mutations are shown as disrupting allosteric communication/dynamic coupling (blue), the structural integrity or residue packing within the ATPase domains cores (orange); ATP binding (purple); DNA binding (cyan). The yeast Rad26 residues are labeled in blue while the residue numbering in human CSB sequence is shown in black in parentheses. Rad26 dynamic communities are colored in green, purple, and light blue. **b** In vitro DNA translocase activities of *S. pombe* CSB ortholog Rph26 mutated proteins measured by a chromatin remodeling assay. The Rhp26 remodeling activity is characterized by analyzing the pattern changes of the restriction enzyme HaeIII cleavage in nucleosomes assembled on a 3 kbp plasmid DNA. The three mutations at positions corresponding to CS disease mutants in the human CSB sequence dramatically reduce chromatin remodeling activity. Quantitation of enzyme activities are shown in the right panel. The experiments were repeated at least three times independently with similar results. Mean values and standard deviations are shown as column bars. *p*-values were calculated using Two-tailed Student’s *t* test (*p*-values N475D/4.89E−08, V744G/0.0297 and L774P/5.46E−07, respectively). Source data are provided as a Source Data file.

Our Pol II–Rad26 model and dynamics simulations also aid in interpreting the effects of disease mutations associated with Cockayne syndrome. Based on the severity of symptoms and age of onset, CS patients are classified into three types: moderate type I CS, with the first symptoms appearing from the end of the first year of life and mortality occurring prior to the age of 20; early-onset and/or severe type II CS; and late-onset type III CS. Cerebro-oculo-facio-skeletal (COFS) syndrome has also been used to describe a very severe form of the disorder with disease onset at the prenatal stage^25,52^. First, we mapped the positions of 19 missense CSB mutations to the equivalent residues of our Pol II–Rad26 model after aligning the human CSB and yeast Rad26 sequences (Fig. 6a, Supplementary Fig. 6, Supplementary Table 2, and Supplementary Movie 1). Disease mutations impact primarily the CSB ATPase core (9 in the RecA1 and 10 in the RecA2 domain).

Sequence comparison of the C-terminal ends between human CSB (residues 1010–1493) and yeast Rad26 (residues 861–1085) shows < 30% sequence identity, which complicates mapping of disease mutations for this region. Three mutations found in the CTD sequences (P1042S, P1095R and R1213G) fall outside the extent of our structural model and could not be mapped. Mutations P1042S, P1095R and R1213G (and all mutations thereafter) are numbered for the human CSB protein.

All but two mutants are positioned in or near the seven conserved Rad26 motifs. Most are found at the ends of helices or in loop regions, indicating their impact on protein dynamics or on the stability of RecA1/RecA2 secondary-structure elements. Importantly, mutations preferentially localize at the interfaces of three Rad26 dynamic communities—F (RecA1), C (RecA2), R (CTD latch). Five are at the F–C community interface (L536W, D646N, N680D, Q953K and L987P); two at the F–R interface (W686C and S687L) and one at the juncture of all three communities (L987P). Additionally, two mutants map onto the functionally important HD2-2 region. Mutations at community interfaces may impact Rad26 dynamics or disrupt key interactions of the ATPase modules, affecting nucleotide binding during ATP-hydrolysis. Mutations may also act allosterically. To address allosteric communication through Rad26, we generated correlation-weighted dynamic networks for the complex in apo, ATP and ADP states and used these to compute suboptimal paths connecting the nucleotide-binding site and the DNA-insertion helix (Supplementary Fig. 7). Suboptimal paths encode dynamic residue correlation from MD and are, thus, indicative of the allosteric pathways traversing the Pol II–Rad26 complex. In the nucleotide-bound states, ATP or ADP serves as a linchpin holding the ATPase core together. Consequently, most suboptimal paths pass directly through the RecA1–RecA2 interface (via helices α23 and α24), across the RecA2 central beta sheet (strands β7, β8, β10, β11, and β12), along the α20 helix, and eventually converge on the DNA-insertion helix (α19). By contrast, in the apo state suboptimal paths are diverted away from the RecA1–RecA2 interface towards dynamic community R, following a series of alpha helices—α16, α17, α31, α25, and α19. Both sets of paths are important for Rad26 dynamics during the ATPase cycle. Strikingly, 70% (14 out of 19) of CS mutations line along these dominant allosteric pathways. Out of the 14 mutants seven (D532G, S687L, N680D, W686C, L987P, V773G and Q953K) are distal from both the ATP-binding site and DNA and, therefore, exert their effect by disrupting allosteric communication through the ATPase core (Fig. 6 and Supplementary Fig. 7). This finding highlights the crucial role of dynamic communication for energy transduction through the Rad26 ATPase core.

Another distinct set of mutations localize to the domain cores (Fig. 6a), likely impacting protein stability or compromising the integrity of key secondary-structure elements (G528E, R670W, I543F, W851R, L871P, L875P, A926P). Mutations to proline are particularly disruptive as they redirect the protein backbone. Finally, three mutants (W936C, P934T and R975W) directly impact DNA binding. W936C in motif Va prevents stabilization of the DNA upstream fork, P934T redirects the protein backbone near DNA, and R975W creates a clash with DNA.

We targeted six CSB missense mutations for experimental validation using restriction enzyme accessibility assays in the yeast CSB ortholog Rhp26. Four mutants were classified as important for allosteric communication: N680D positioned at the F–C community interface; W686C and S687L at the F–R interface; and L987P at the junction of the F, C and R communities. Two mutations, W851R and V957G, were positioned in the domain cores likely affecting protein stability or packing of secondary-structure elements. Three mutants (W686C, S687L, W851R) failed to express as soluble proteins, indicating that these residues are important for proper folding and stability Rhp26, which is consistent with our functional annotation of mutants in Supplementary Table 2. We then measured the capabilities of remaining three yeast CSB mutants in remodeling nucleosome arrays, which requires their DNA translocation activities. All three mutants showed reduced activities in comparison with WT (Fig. 6b) in the order WT > V957G > N680D > L803P. Intriguingly, the observed ordering correlates well with clinical phenotype: e.g. L803P (Type II, COFS) has more severe phenotype than N680D and V957G (Type I). The V957G is the least severe among the mutants we tested, which is consistent with the structural observation that this residue localizes at the juncture of a helix and loop of RecA2 that are important for allosteric communication. N680D and L987P greatly impede CSB translocation activities, highlighting the functional importance of the F-R community interface.

We also classified several deletion/insertion mutations causing severe CS **(**Supplementary Fig. 6c and Supplementary Table 2**)**. Motif I and III are responsible for ATP hydrolysis and deletions R467-R562del, Y510-R562del, F665-Q723del, and W589del directly impact ATPase activity. Deletions V724-Q762del and V763-Q794del occur in HD2-1 region and disrupt DNA binding. The M752-Q762del deletion localizes to the hinge region between RecA1 and RecA2, affecting dynamics and the ability of the ATPase modules to close in response to nucleotide binding. One insertion, K538-T539delinsKNVF, disrupts motif I impairing ATPase activity^53^. E608-Q723del is a very extensive deletion affecting the entire ATPase core. Predictably these deletion/insertion mutations are known to cause the most severe type II CS phenotype.

## Discussion

The wide variety of lesions processed by the transcription-coupled repair pathway has led to the evolution of a remarkably complex protein machinery. To unravel the precise functional roles of CSB (or Rad26) in this machinery, we built suitably complete structural models of Pol II–Rad26 complexes in apo, ATP- and ADP-bound states. We then employed extensive molecular dynamics simulations and novel graph-theoretical methods to analyze the functional dynamics of these assemblies.

**Fig. 7:**
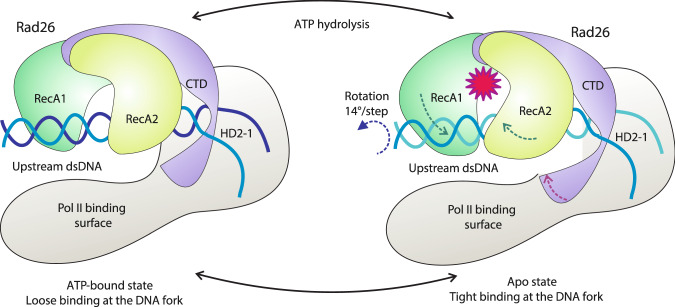
Mechanism of Rad26-assisted Pol II forward translocation on a damaged DNA template. The RecA1 and RecA2 domains are shown in dark and light green, CTD and the insertion helices are shown in purple, Pol II in gray, the DNA strands in light and dark blue. The directions of the swing motions of the ATPase domains and the rotation of the upstream DNA duplex are depicted by dashed arrows. The red star indicates the ATP hydrolysis event.

We support a mechanism wherein Rad26 binds to the upstream DNA duplex of a stalled Pol II, redirects the path of DNA and recognizes the edge of the transcription bubble through specific interactions, involving conserved structural elements—HD1, HD2-1, HD2-2 and the CTD. Nucleotide-driven closure and opening of the ATPase modules allow the Rad26 molecular motor to shift and rotate the upstream DNA duplex while alternating tight and loose DNA binding of the RecA2 domain at the transcription bubble fork causes pulling on the template strand (Fig. 7). Thus, our models reveal a possible structural basis for Rad26-mediated DNA remodeling to assist Pol II progression on a damage-containing DNA template. While such forward motion along the DNA template may aid transcriptional bypass of less bulky DNA lesions, it may also result in extreme Pol II stalling upon encounter of a bulky lesion. In turn, this may trigger recruitment of downstream NER factors—CSA, UVSSA and TFIIH. In this way, Rad26 provides a mechanism for lesion discrimination in the early steps of transcription-coupled repair. For this model to be operational, the Rad26 molecular motor must be firmly attached to the rest of the Pol II machinery. Correspondingly, we find that the previously unmodeled CTD and NTD domains play a key role in anchoring Rad26 to Pol II and establishing a productive orientation with respect to the transcription bubble.

Furthermore, dynamic network analysis of the vast conformational ensembles from MD led to the discovery of a global allosteric network extending from Rad26 along the circumference of the Pol II central cavity. Thus, we support an allosteric mechanism for CSB/Rad26 to power distal conformational changes, affecting the opening/closing dynamics of Pol II and the DNA transcription bubble. Such conformational changes may allow Pol II to bypass lesions in the transcribed strand. We also mapped Cockayne syndrome disease mutations onto our model and examined their positioning in the context of dynamic communities and allosteric pathways identified from network analysis. This allowed us to predict their effects in disrupting local structure and dynamics, providing insights into disease etiology.

Collectively, our results impact understanding of CSB (Rad26) as a central constituent of the transcription-coupled repair machinery and shed light on fundamental mechanisms by which harmful DNA lesions are detected and removed from the transcribed genome.

## Methods

### Model building

To model Rad26, we used the available Pol II–Rad26 cryo-EM density (EMDB accession code: EMD-8735)^28^. The Rad26 NTD (residues 94–228) had no known structural homologs and was, therefore, built de novo. The structures (229–297 and 559–577) of 5VVR were rebuilt based on the EM density and secondary-structure prediction (Supplementary Fig. 2c and Supplementary Table 3). We used the GeneSilico metaserver for consensus secondary-structure prediction^54^ and applied the results to establish the sequence register in the EM density. Individual secondary-structure fragments were constructed using COOT^55^ to generate a backbone only model by tracing the EM density. The resulting polypeptide chain segments were connected by extending the main-chain trace. Side chain orientations were built and manually adjusted based on the electron density. Bulky residues such as phenylalanine, tyrosine, tryptophan, and arginine were instrumental in validating model construction and sequence registration. To model the Rad26 CTD (residues 798-860), the structures of the yeast *T. thermophilus* ISWI (PDB ID: 5JXR^41^), *S. cerevisiae* SWI2/SNF2 (PDB ID: 5X0Y^40^), and *M. thermophila* SNF2 (PDB ID: 5HZR^39^) were used as templates to construct the Rad26 CTD homology structure. The missing residues from the CTD (861–1085) were traced in the original EM density and built de novo. We then built the entire Rad26 by docking the newly constructed NTD and CTD into the density. The docked model was then manually adjusted to the cryo-EM density using COOT^55^. The final Rad26 structure was added to Pol II from the 5VVR structure to produce the complete Pol II–Rad26 complex, which was subsequently refined in a 4-ns molecular dynamics flexible fitting (MDFF) simulation^56–58^. The MDFF biasing potential was applied with a scaling factor ξ of 0.2. At the end of our protocol, we performed real space refinement to the EMD-8735 cryo-EM density using the PHENIX package^59,60^, Finally, we constructed Pol II–Rad26 complexes in the ATP- and ADP-bound state by placing the ATP or ADP ligands into the Rad26 nucleotide-binding pocket based on an yeast SNF2 structure crystallized with ADP-BeFx (PDB ID: 5Z3V)^43^. Mg^2+^ ion coordination was also based on the yeast SNF2 structure. The evaluate the map-to-model fit we computed cross-correlation coefficients (CCC) with UCSF Chimera for Rad26 only and for the entire Pol II–Rad26–DNA assembly. The CCC values were 0.66 for Rad26 and 0.77 for the Pol II–Rad26 complex, respectively.

### Molecular dynamics

To address the Pol II–Rad26 functional dynamics we performed extensive molecular dynamics simulations on the Summit machine at the Oak Ridge Leadership Computing Facility. All simulation systems were set up with the TLeap module of AMBER^61^ and solvated with TIP3P water molecules^62^ in a box with a minimum distance of 12.0 Å from the protein to the edge of the simulation box. The simulation boxes for the apo, ATP- and ADP-bound systems had dimensions of 178.3 × 189.1 × 186.2 Å^3^, 182.4 × 192.2 × 190.3 Å^3^ and 182.2 × 192.3 × 190.3 Å^3^ and contained 630,217, 579,191 and 579,192 atoms, respectively. ADP and ATP were obtained from the AMBER parameter database^63^. Counterions were added to neutralize the total charge of the protein complex and reach 150-mM NaCl concentration to mimic physiological conditions.

Energy minimization was conducted for 3000 steps with fixed protein backbone atoms and for an additional 1500 steps with harmonic restraints on the backbone atoms (*k* = 10 kcal mol^−1^ Å^−2^). The temperature of the simulated systems was then gradually increased to 300 K over 500 ps of dynamics in the NVT ensemble. Positional restraints were imposed on all heavy atoms (*k* = 10 kcal mol^−1^ Å^−2^). Equilibration was continued for another 5 ns in the NPT ensemble, and positional restraints were gradually released to fully equilibrate the systems. Production runs were conducted with a 2-fs timestep in the NPT ensemble (1 atm and 300 K) for ~11 μs for each of the Pol II–Rad26 complexes (apo, ATP- and ADP-bound). The smooth particle mesh Ewald (SPME)^64^ electrostatics was employed with 10 Å cutoff for short-range non-bonded interactions. The simulations were performed with the CUDA version of the Amber18 PMEMD code using the Parm14SB force field^65^ and bsc1 modifications^66^ to the nucleic acid parameters. In total, 11 independent production runs of ~1 μs length were completed for each system (apo, ATP and ADP). We selected the last ~280 ns of each independent trajectory based on root-mean-square deviation (RMSD) convergence. RMSD values were computed over the protein Cα atoms and nucleic acid P atoms. More than 100,000 conformations per functional state were selected for clustering analysis to identify the dominant conformations and for dynamic network analysis with the dCNA code^44^. All figures were generated using UCSF Chimera^60^.

### Difference contacts network analysis

In the difference contacts network analysis method (dCNA), a consensus network was first constructed for all functional states (apo, ATP- and ADP-bound). The Girvan-Newman algorithm was then applied on the consensus network to subdivide the assembly into dynamic communities. Once the community structure was identified, a second step involving subtraction of contact probability maps was carried out to detect changes in between the functional states. Contact maps were generated with the MDTraj package^61^ using the last 280 ns from the MD trajectories of each Pol II–Rad26 functional state (apo, ATP- and ADP-bound). To obtain the consensus network, contact maps for the ADP and ATP states were subtracted from the apo map. Edges were drawn between nodes and assigned a weight of 1.0 if the change in contact probability was > = 90%, indicating persistence across all functional states. In order to derive the community structure from the consensus network, we relied on a non-weighted version of the Girvan-Newman algorithm, available in the Python package NetworkX. The consensus network was continually subdivided using Girvan-Newman^62^ until the difference in modularity between subsequent partitions was <0.001. The final partition yielded 22 distinct communities with a modularity of 0.91. Finally, the change in contact probability between the consensus communities was decomposed into individual transitions representing the ATP hydrolysis cycle (APO$\to$ATP, ATP$\to$ADP, ADP$\to$APO). This was achieved by subtracting the relevant contact maps from each other (i.e. ATP–APO, ADP–ATP, APO–ADP) to obtain three distinct difference networks. We then focused on the overall change in contact probability between the consensus communities in each transition. The overall change (ΔP) was determined by summing the individual differences between two distinct communities,

1 $$\Delta {P}_{AB}=\sum {p}_{ij},{{{{{\rm{if}}}}}}\,i\in A\,{{{{{\rm{and}}}}}}\,j\in B$$

Note that we only consider the difference in contact probability between residue *i* and *j* (*p*_*ij*_) if they reside in two different communities *A* or *B*. Since *p*_*ij*_ can take on both positive and negative values, the value of the overall change is either negative (indicating loss in interactions), positive (indicating gain in interactions) or zero (indicating no change).

### Suboptimal paths

To compute suboptimal paths, we relied on dynamic network analysis. In dynamic networks, protein and DNA residues are represented as nodes with edges that connect nodes in persistent contact. Persistent contacts, in this case, were defined as having one or more heavy atoms within a distance of 4.5 Å of each other for more than 75% of the MD trajectory. Edge weights were then calculated using cross-correlation,

2 $${c}_{{ij}}=\frac{\left\langle \left({r}_{i}-\left\langle {r}_{i}\right\rangle \right)\cdot \left({r}_{j}-\left\langle {r}_{j}\right\rangle \right)\right\rangle }{{\left\langle {\left|{r}_{i}-\left\langle {r}_{i}\right\rangle \right|}^{2}\right\rangle }^{1/2}{\left\langle {\left|{r}_{j}-\left\langle {r}_{j}\right\rangle \right|}^{2}\right\rangle }^{1/2}}\,$$

where ***r*** is the positional vector of residues *i, j* and $\left\langle {{{{{\boldsymbol{r}}}}}}\right\rangle$ is the time-evolved average of the positional vector computed from the MD trajectory. The final edge weights are then transformed through,

3 $${w}_{{ij}}=-{{{{{{\rm{ln}}}}}}}\,(|{c}_{{ij}}|)$$

We determined networks for all three functional states (apo, ADP, ATP) using the last 280-ns of each trajectory. From these networks, the first 5000 suboptimal paths were computed using the SOAN method^67^. The source and target residues were R612 and D469, respectively, and were selected based on their positioning within the Rad26 structure. This choice was aimed at identifying allosteric communication paths originating in RecA1 (proximal to the Rad26 active site) and leading to the insertion helix at the edge of the DNA transcription bubble. In the SOAN method, all nodes two neighbors away from the optimal path were considered when reducing the original graph to improve the efficiency of the suboptimal path determination.

### Restriction enzyme accessibility assays

To evaluate the significance of individual residues, we used Rhp26 as a model due to the technical challenge of expressing and purifying pure Rad26 proteins (wild-type and mutants). Expression and purification of Rhp26 wild type and mutants were performed essentially as previously described^30^. Restriction enzyme accessibility assays were used to characterize the chromatin remodeling activity of Rhp26 (DNA translocase activity on chromatin template) and performed as described previously with minor modifications. Briefly, chromatins were reconstituted by the gradient salt dialysis method by using *Xenopus laevis* core histones and an ∼3-kb plasmid DNA. Then, 200 ng of chromatin were gently mixed with Rhp26 in 1X NEB cut smart buffer containing 3 mM ATP and 5 mM MgCl_2_. The remodeling reaction was performed at 30 °C for 1 h followed by adding 15 U of HaeIII restriction enzyme (NEB) to digest the remodeled chromatin. After digestion for 1 h at 27 °C, samples were deproteinized, and DNA was purified, resolved by 1% agarose gel, and visualized by Gel Red (Biotium) DNA staining.

### Reporting summary

Further information on research design is available in the Nature Research Reporting Summary linked to this article.

## Supplementary information

Supplementary Information

Description of Additional Supplementary Files

Supplementary Movie 1

Supplementary Movie 2

Supplementary Movie 3

Reporting Summary

## Data Availability

The data that support the findings of this study are available from the corresponding authors upon reasonable request. The models of Pol II–Rad26 in apo, ATP-bound and ADP-bound states have been deposited in the ModelArchive database with DOI accession codes: 10.5452/ma-hxt70, 10.5452/ma-bd9wm and 10.5452/ma-5liiv, respectively. Source data are provided with this paper.

## Source data

Source Data
