# Supplementary material for: Mechanism of Rad26-assisted rescue of stalled RNA polymerase II in transcription-coupled repair
Source: Nat Commun. 2021 Dec 1;12:7001. doi: 10.1038/s41467-021-27295-4 (PMC8636621; doi:10.1038/s41467-021-27295-4)
Supplement: Supplementary file 1 — Supplementary Information [file 41467_2021_27295_MOESM1_ESM.pdf]

SUPPLEMENTARY FIGURES

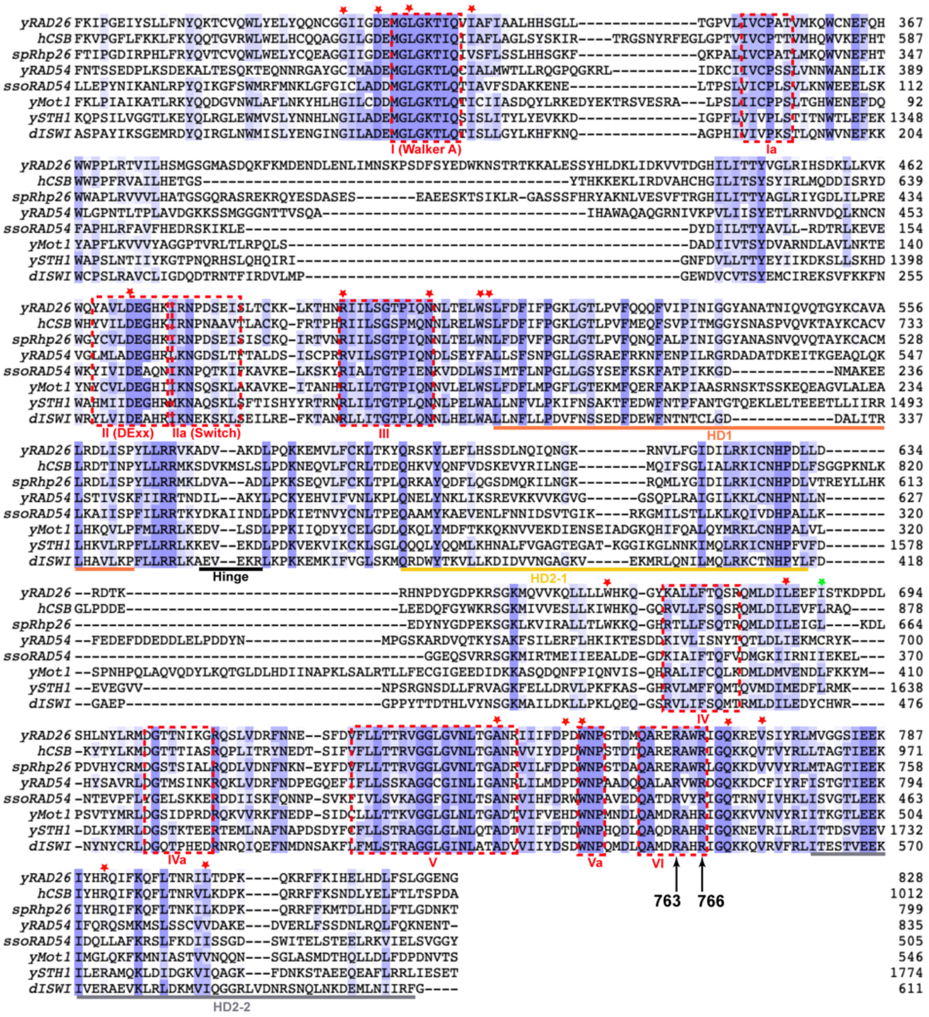

Supplementary Figure 1. Sequence alignment of Rad26 ATPase core (catalytic domain) with SWI2/SNF2 enzymes. Canonical super family 2 (SF2) helicase motifs are highlighted by red dashed-line boxes. Additional conserved regions are shaded light blue (moderately conserved) or dark blue (strongly conserved). The HD1, HD2-1, HD2-2, and hinge region between the two RecA domains are highlighted by horizontal lines in orange, red, gold and grey, respectively. Positions of missense disease mutations associated with Cockayne syndrome are indicated by red stars. Abbreviations: *yRAD26*, *Saccharomyces cerevisiae* Rad26; *hCSB*, human Cockayne syndrome protein B; *sp*Rhp26, *Schizosaccharomyces pombe* Rhp26; *yRAD54*, *Saccharomyces cerevisiae* Rad54; *SsoRAD54*, *Sulfolobus solfataricus* SWI2/SNF2 homolog; *yMot1*, *Saccharomyces cerevisiae* Mot1; *ySTH1*, *Saccharomyces cerevisiae* STH1; and *dISWI*, *Drosophila melanogaster* imitation SWI.

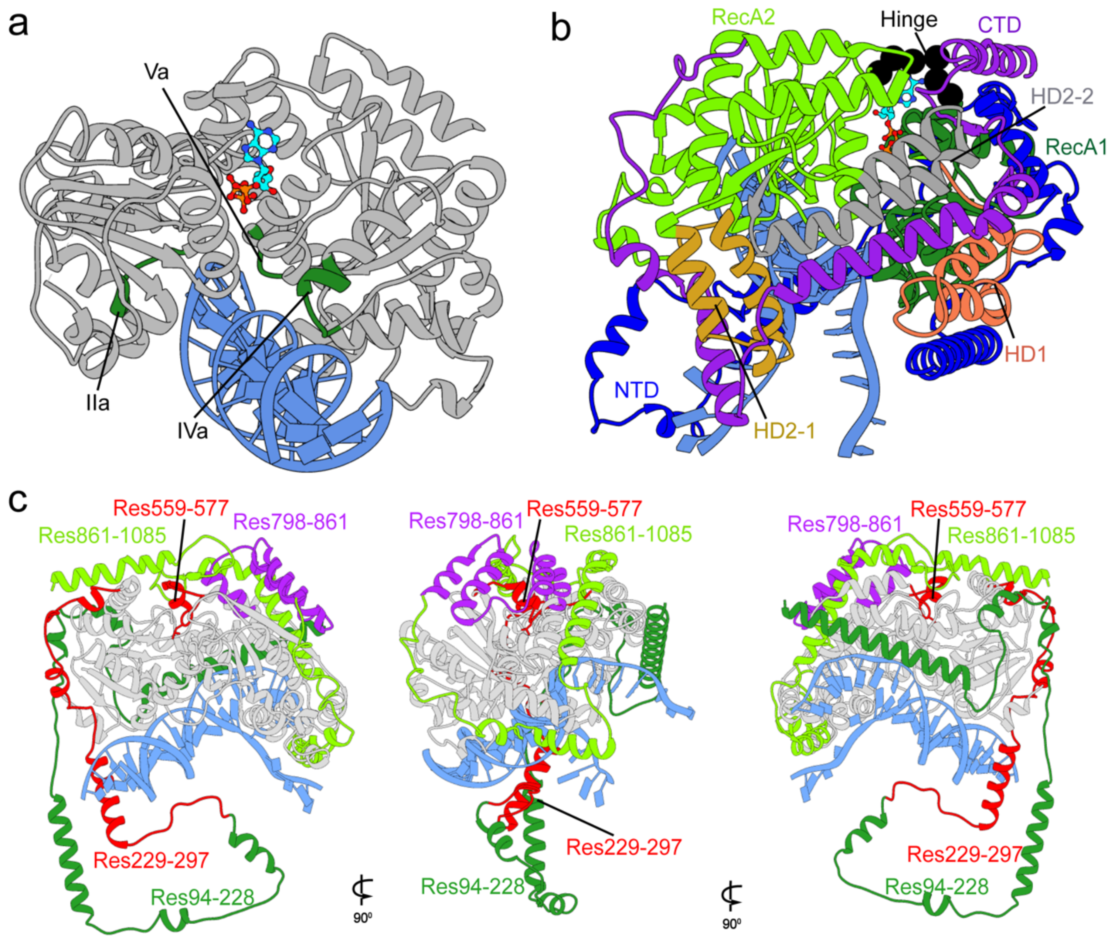

Supplementary Figure 2. DNA binding mode of Rad26. a. Conserved motifs involved in Rad26-DNA binding. **b.** Structural elements of Rad26 - The N-terminal region (NTD), RecA1, HD1, hinge, RecA2, HD2-1, HD2-2, and the C-terminal region (CTD) are shown in blue, dark green, orange, black (sphere), light green, tan, and grey, respectively. **c.** Newly modeled Rad26 regions. Structural elements modeled *de novo* are shown in dark green and light green. Structural elements revised and rebuilt from the previous cryo-EM model (5VVR) are shown in red. Structural elements constructed by homology modeling are shown in purple.

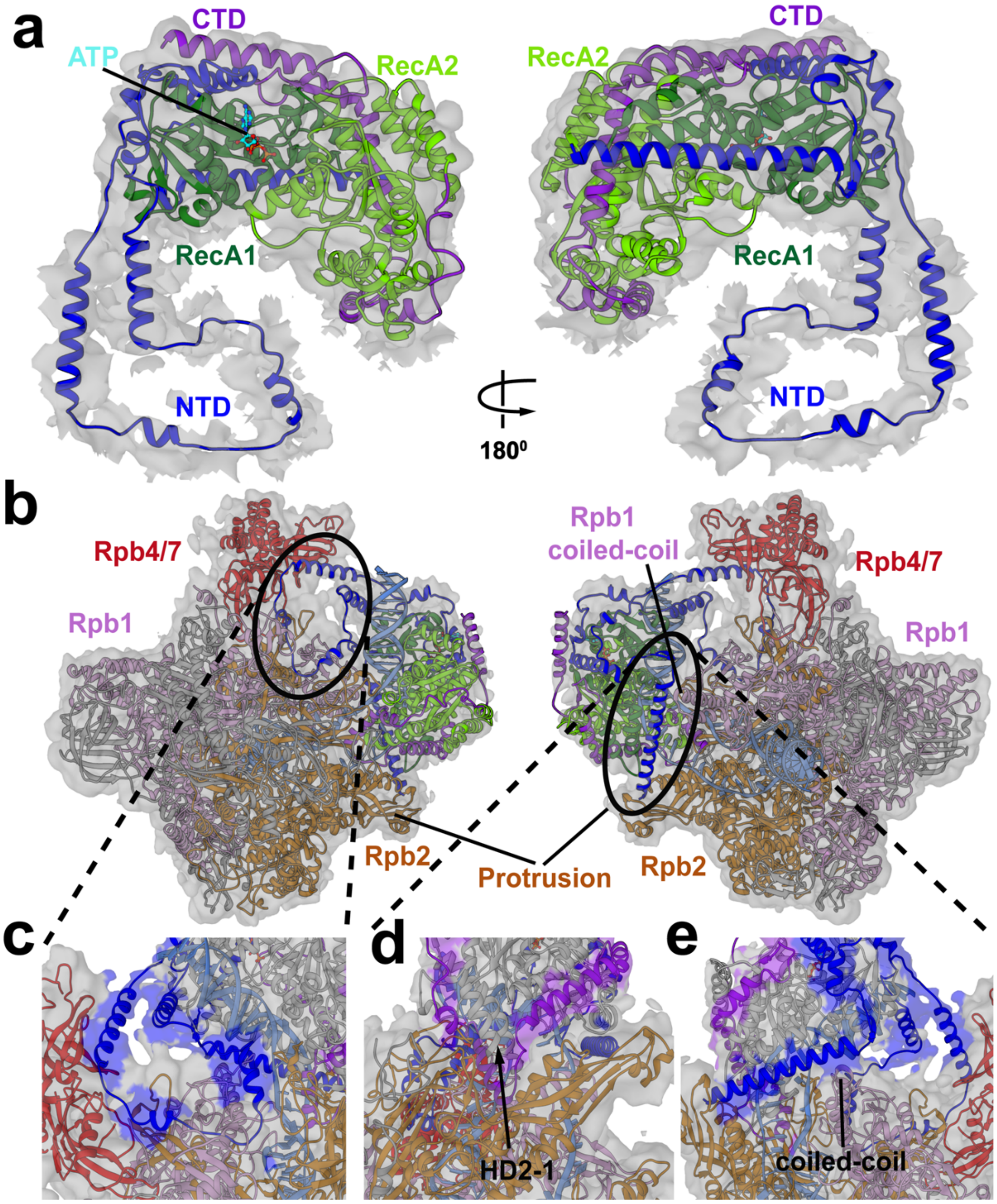

Supplementary Figure 3. EM densities and corresponding atomic models for the Pol II-Rad26 assembly. a, Rad26 domains fitted into the segmented Pol II-Rad26 EM density (EMD-8735). **b,** Overall fit of the Pol II-Rad26 model to the EM density (EMD-8735). Rpb1, Rpb2 and Rpb4/7 are in light purple, brown and red, respectively. Circles demark zoomed regions in c.-e. **c**, The Rad26 NTD interacts with Rpb1 (light purple), Rpb2 (brown) and Rpb4/7 (red). **d**, Rad26 CTD and HD2-1 insert into the DNA bubble and interact with Rpb2. **e**, Rad26 NTD interacting with the Rpb2 protrusion and the Pol II clamp coiled-coil loop. The densities of Rad26 NTD and CTD are highlighted in blue and purple.

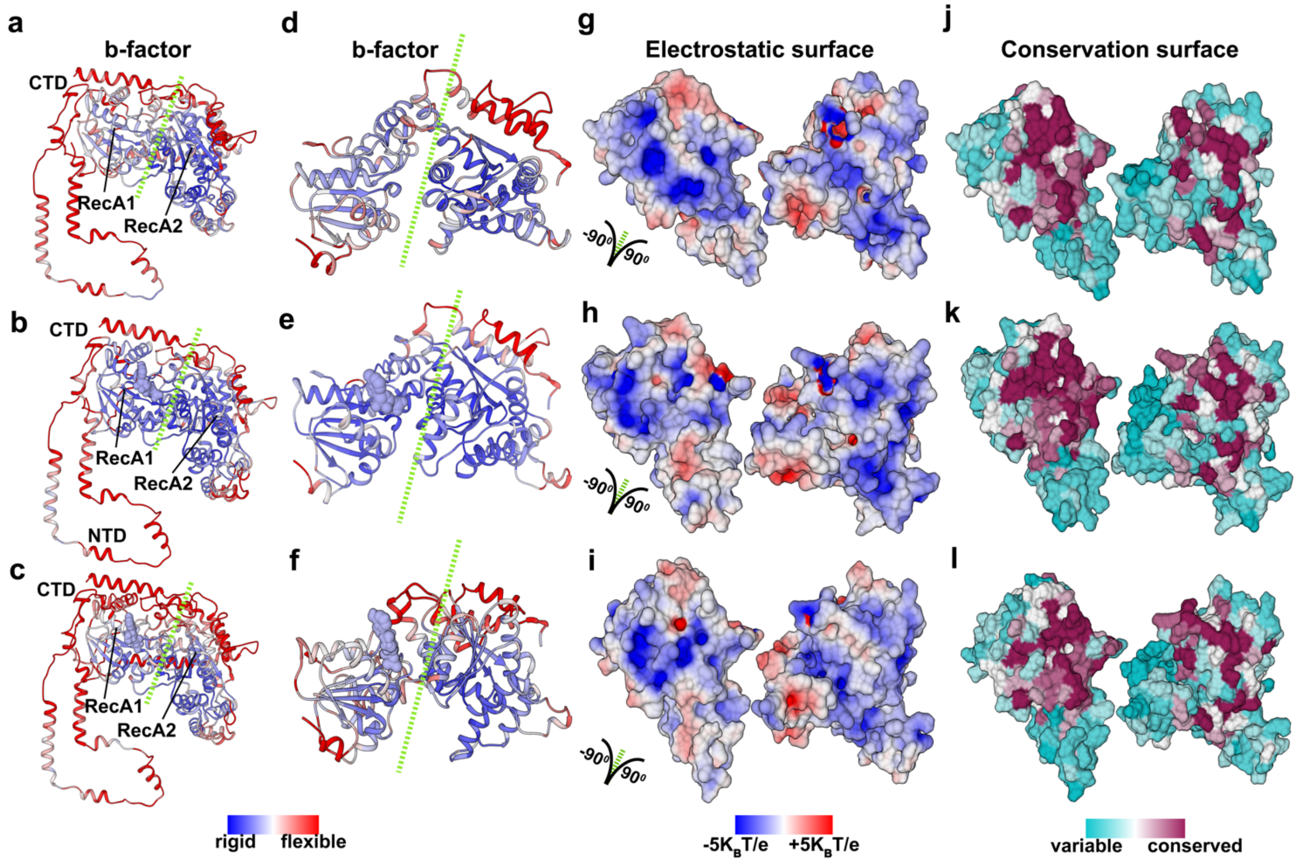

Supplementary Figure 4. Comparison of the apo, ATP and ADP RecA1-RecA2 interfaces in terms of computed B-factors, electrostatics and conservation. a-f, Computed B-factors mapped onto the Rad26 structural model with values colored from high (red) to low (blue) for **a.,** apo; **b.,** ATP-bound; and **c.,** ADP-bound model. The interface of RecA1 and RecA2 is indicated by a green dashed line. **d-f** panels show zoomed-in views of the corresponding RecA1-RecA2 interfaces. **g-i,** Surface electrostatics of the RecA1 and RecA2 interfaces in the following models: **g,** apo; **h,** ATP-bound and **i,** ADP-bound. Electrostatic potential mapped onto the molecular surface and colored from red (negative) to blue (positive). **j-l**, Conservation maps of the RecA1 and RecA2 interfaces are shown for the following models: **j,** apo; **k,** ATP-bound and **l,** ADP-bound. Highly conserved regions are shown in brown, semi-conserved regions in white, and non-conserved regions in cyan. Conservation maps were based on alignment of the following sequences: *Saccharomyces cerevisiae* Rad26, human Cockayne syndrome protein B, *Saccharomyces cerevisiae* Rad54, *Sulfolobus solfataricus* SWI2/SNF2 homolog, *Saccharomyces cerevisiae* Mot1, *Saccharomyces cerevisiae* STH1, and *Drosophila melanogaster* imitation SWI.

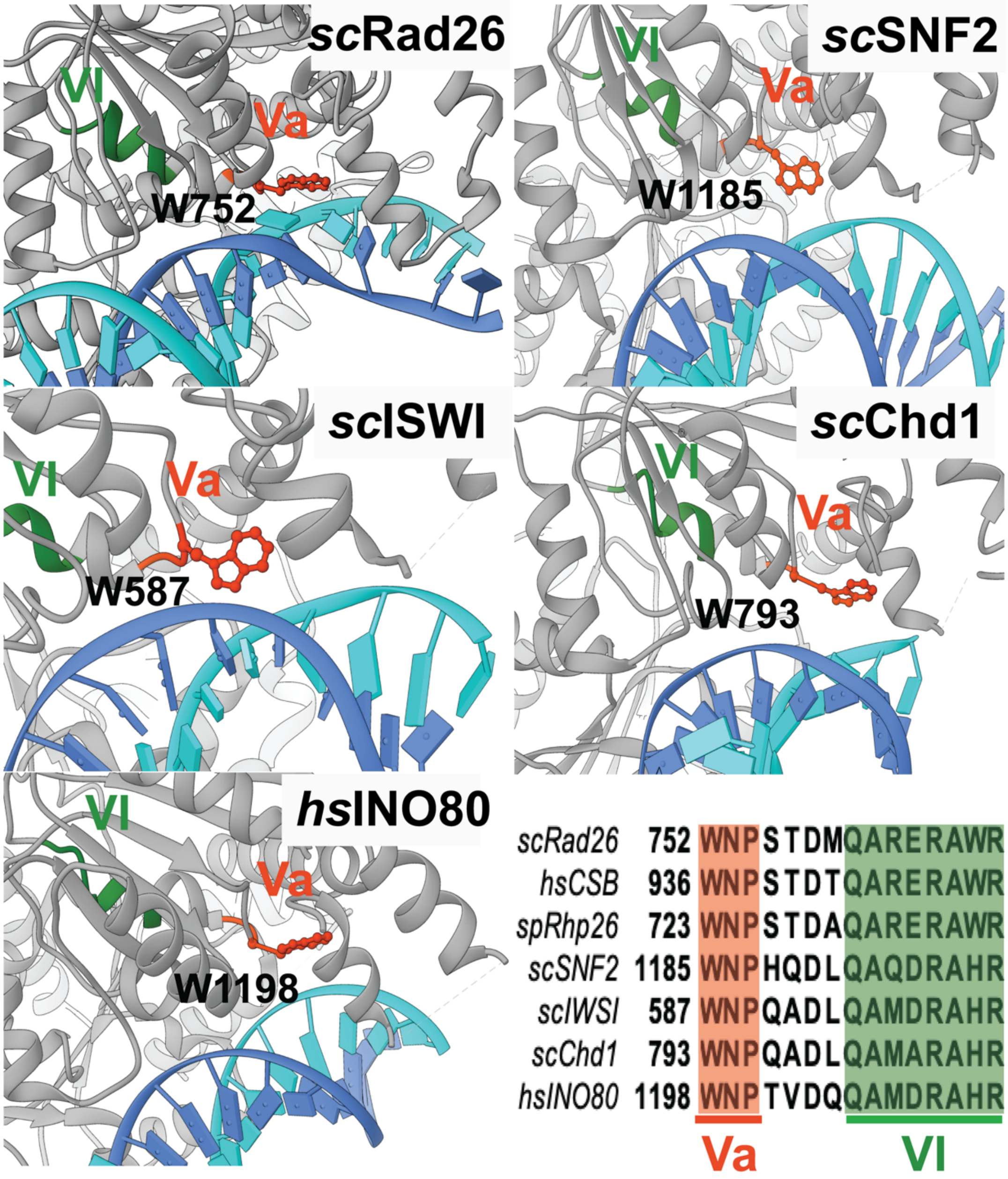

Supplementary Figure 5. Rad26 W752 residue is conserved across the CSB protein subfamily and chromatin remodelers. The DNA binding mode of the conserved tyrosine residue (red) is shown for the following proteins: *scRAD26*, *Saccharomyces cerevisiae* Rad26; *hCSB*, human Cockayne syndrome protein B; *spRhp26*, *Schizosaccharomyces pombe* Rhp26; *scSNF2*, *Saccharomyces cerevisiae* SNF2; *scChd1*, *Saccharomyces cerevisiae* Chd1; *scIWSI Saccharomyces cerevisiae* imitation of switch; and *hsINO80*, *Homo sapiens* INO80. The last panel shows the sequence alignment of motifs Va and VI of the ATPase core.

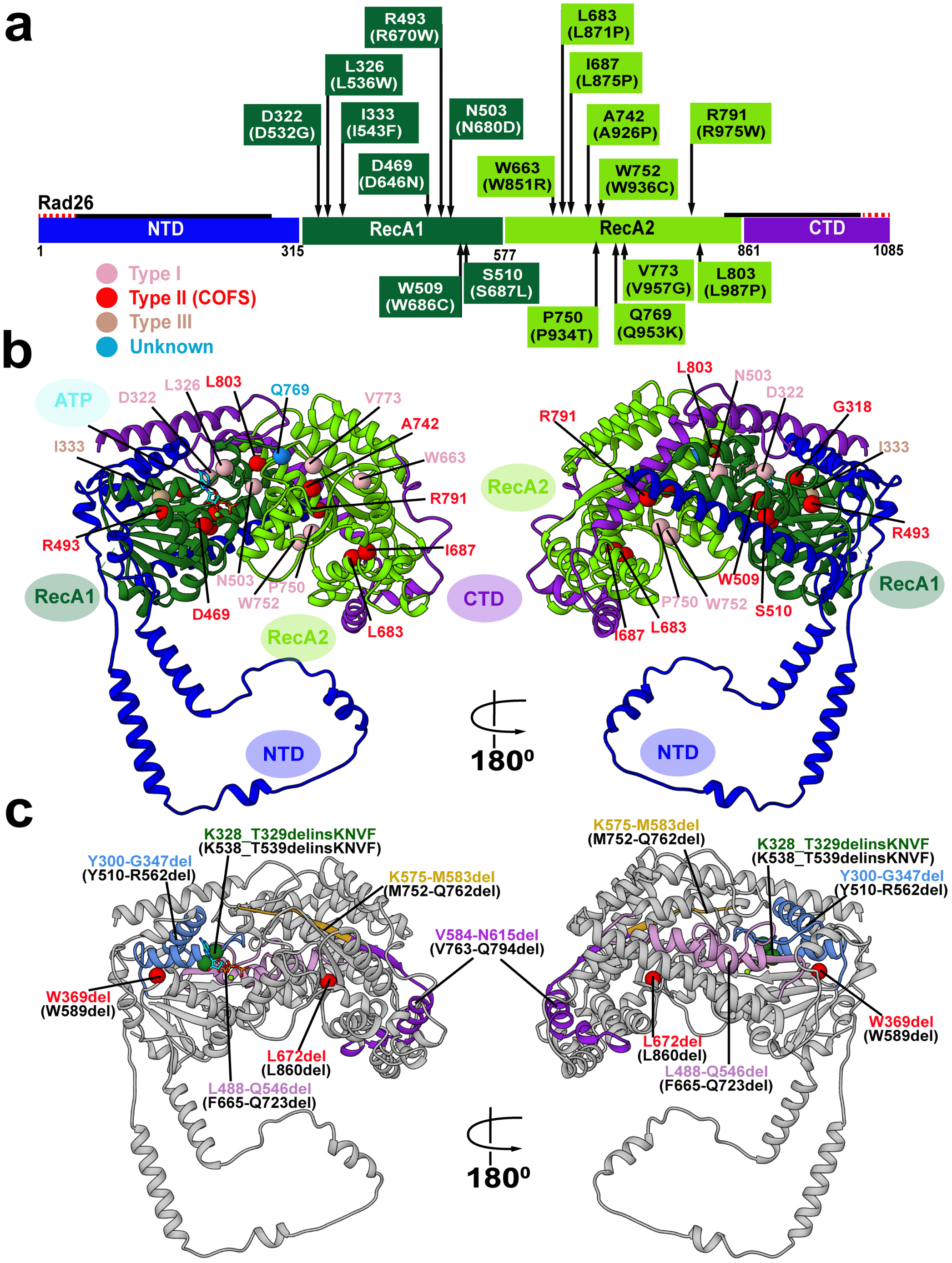

Supplementary Figure 6. Mapping of missense Cockayne syndrome mutations onto Rad26. **a**, Cockayne syndrome point mutations mapped onto the Rad26 primary sequence. The top labels indicate the yeast Rad26 mutations and a colored by disease phenotype. The corresponding human CSB mutations are labeled below in black and enclosed in parentheses. **b**, Positions of Cockayne syndrome point mutations are shown as pink (Type I), red (Type II), tan (Type III) and light blue (Unknown) spheres. **c**. Mapping of deletion/insertion mutations onto the Rad26 model.

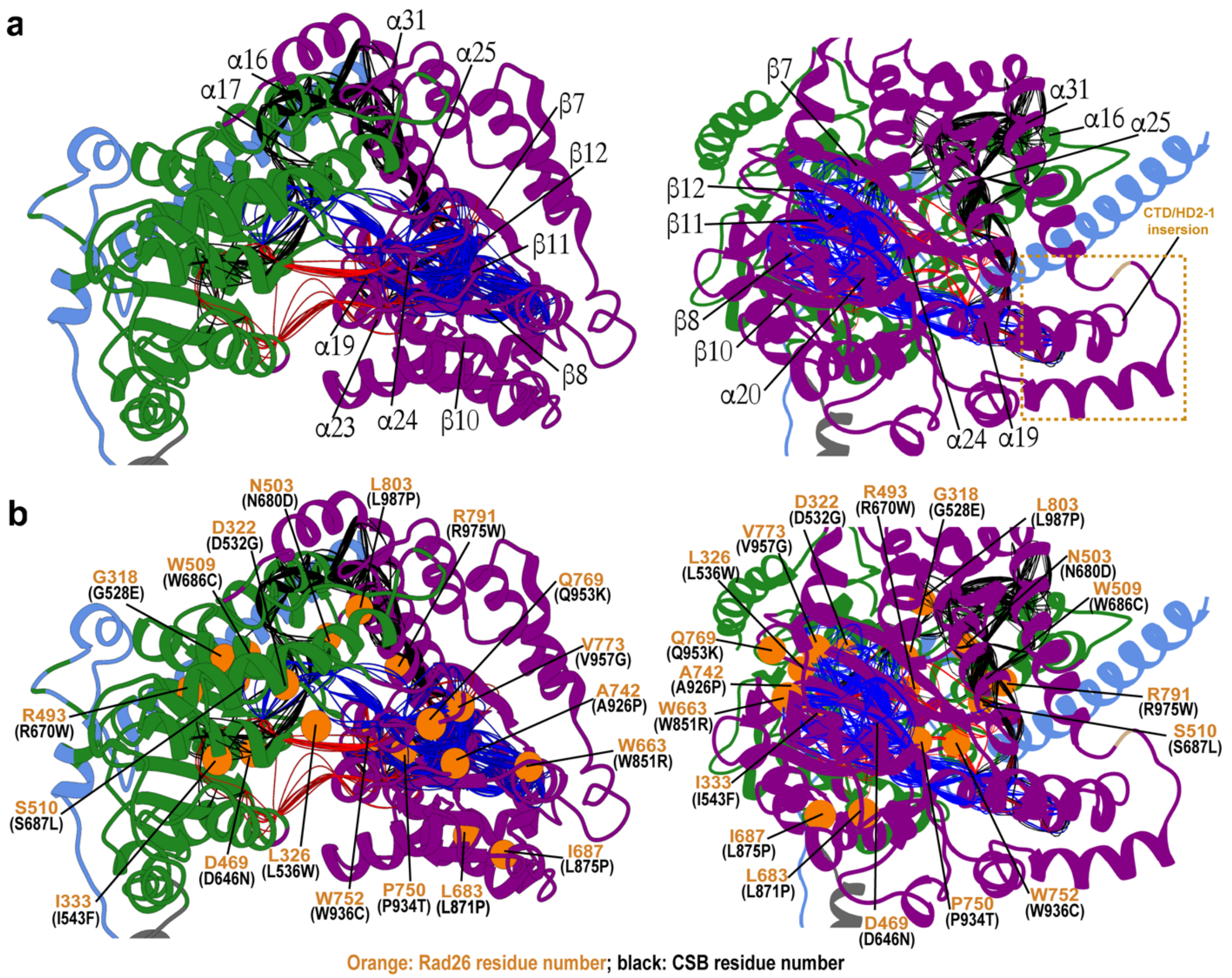

Supplementary Figure 7. Suboptimal paths connecting the ATP-binding site and the DNA fork insertion helix of Rad26. a, Paths (colored tubes) traverse the Rad26 core from the ATP site toward the CTD/HD2-1 insertion (dotted box). Secondary structure elements that fall along the dominant paths are labelled. **b,** Patient mutations superimposed on the suboptimal path network and labeled by position in the Rad26 sequence (orange labels) or the human CSB sequence (black labels). Rad26 is colored by dynamic community, while the paths are colored by functional state: black denotes apo, red denotes ATP-state and blue denotes ADP-state suboptimal paths.

SUPPLEMENTARY TABLES

Supplementary Table 1. The buried surface area of NTD and CTD with RecA1, RecA2, Pol II, and DNA

	Buried Surface Area (Å^2)
DNA-NTD	261.44
DNA-CTD	275.01
RecA1-NTD	3611.65
RecA1-CTD	1040.95
RecA2-CTD	3661.41
Pol II-RecA core	681.00
Pol II-NTD	937.21
Pol II-CTD	712.00

Supplementary Table 2. Human disease mutations in CSB

Mutation (CSB)	Rad26	CS[c] type	C ID[d]	Inferred functions[f]	Reference, Activity, Structural Analysis, Inferred Change
G528E	G318	CS II[h]	F	N-end of motif I	DOI: 10.1136/jmedgenet-2017-104877<br>G318 is in a beta-sheet within the RecA1 core. Mutation to E introduces charge in a hydrophobic environment, disrupting surrounding hydrophobic cluster. Mutation to E likely destabilizes the RecA1 core.
D532G[b]	D322	CS I[g]	F	Motif I, ATPase function	DOI: 10.1590/1678-4685-GMB-2019-0085<br>DOI: 10.1136/jmedgenet-2017-104877<br>DOI: 10.1371/journal.pone.0113914<br>D322 is within motif I. D to G mutation removes hydrogen bonding with Q301 and a salt-bridge interaction with K328 to destabilize RecA1.
L536W[b]	L326	CS I[i]	F	Motif I, ATP binding, ATPase function Community connector (F and C)	DOI: 10.1590/1678-4685-GMB-2019-0085<br>DOI: 10.1136/jmedgenet-2017-104877<br>DOI: 10.1371/journal.pone.0113914<br>L326 near the RecA1-RecA2 interface directly interacts with the ATP adenine group. L to W mutation obstructs the ATP-binding pocket, prevents ATP binding, destabilizes interdomain (C-F community) interface.
I543F	I333	CS III[f]	F	C-end of motif I, ATPase function	I333 belongs to motif I and is positioned at the end of a helix near F365, W368, and W369. I to F mutation impacts the helix structure and creates clashes with bulky aromatic rings, disrupting the ATPase core.
D646N	D469	CS II	F	Motif II, ATP and MG binding, ATPase function Community connector (F and C)	DOI: 10.1590/1678-4685-GMB-2019-0085<br>DOI: 10.1136/jmedgenet-2017-104877<br>D469 directly coordinates with the active site metal and interacts with the ATP phosphate group through metal in Walker B motif. Mutation to N directly disrupts ATPase activity and the electrostatics of the interdomain interface.
R670W[b]	R493	CS II	F	N-end of motif III, ATPase Function	DOI: 10.1590/1678-4685-GMB-2019-0085<br>DOI: 10.1136/jmedgenet-2017-104877<br>DOI: 10.1002/humu.21154<br>R493 is in a beta sheet at the N-end of motif III. R to W mutation disrupts a hydrogen bonding network formed by the surrounding residues T140, L488, T490 also affecting residue packing in the domain core.
N680D[b]	N503	CS I	F	Motif III, ATPase function Community connector (F and C)	DOI: 10.1590/1678-4685-GMB-2019-0085<br>DOI: 10.1136/jmedgenet-2017-104877<br>DOI: 10.1002/humu.21154<br>N503 is at the RecA1-RecA2 interface. N to D Mutation destabilizes interdomain interface (communities F and C).

W686C[b]	W509	CS II	F	Near C-end of motif III, ATPase function. Community connector (F and R)	DOI: 10.1590/1678-4685-GMB-2019-0085<br>DOI: 10.1136/jmedgenet-2017-104877<br>DOI: 10.1002/humu.21154<br>W509 is near the C-end of motif III and packs against F526. W to C mutation disrupts the aromatic packing in the hydrophobic core.
S687L[b]	S510	CS II	F	Near C-end of motif III, ATPase function. Community connector (F and R)	DOI: 10.1590/1678-4685-GMB-2019-0085<br>DOI: 10.1136/jmedgenet-2017-104877<br>DOI: 10.1002/humu.21154<br>Mutation to L disrupts ATPase core by losing hydrogen bonding with S482 and destabilizes the F-R dynamic community interface.
W851R[a]	W663	CS I	C	Near N-end of motif IV	DOI: 10.1590/1678-4685-GMB-2019-0085,<br>DOI: 10.1136/jmedgenet-2017-104877,<br>DOI: 10.1002/humu.21154<br>W663 is in a helix near R742. Mutation to R destabilizes the helix position due to repulsive charged interactions with the newly created arginine interactions. This destabilizes the RecA2 hydrophobic core.
L871P[b]	L683	COFS	C	Near N-end of motif IV,	DOI: 10.1136/jmedgenet-2017-104877<br>DOI: 10.1002/humu.21154<br>L683 is in the middle of a helix near the N-end of motif IV. L to P mutation distorts the helix, which no longer packs against the neighboring beta sheet, disrupting the hydrophobic core of RecA2.
L875P[a]	I687	CS II	C	Near C-end of motif IV,	DOI: 10.1590/1678-4685-GMB-2019-0085<br>DOI: 10.1136/jmedgenet-2017-104877<br>I687 is positioned in the same region as L683 and mutating it to P has similar effect.
A926P	A742	CS II	C	Motif V	DOI: 10.1136/jmedgenet-2017-104877<br>A742 is in a helix near the C-end of motif IV. A to P mutation redirects the protein backbone and disrupts hydrophobic packing.
P934T[a]	P750	CS I	C	Near N-end of motif Va, DNA binding	DOI: 10.1590/1678-4685-GMB-2019-0085<br>DOI: 10.1136/jmedgenet-2017-104877<br>DOI: 10.1002/ajmg.a.37501<br>R487 is in a loop neighboring the W752 residue. Mutation to P increases main chain rigidity and redirects the loop so that it cannot as easily accommodate DNA binding.
W936C[a]	W752	CS I	C	Motif Va, DNA binding	DOI: 10.1590/1678-4685-GMB-2019-0085<br>DOI: 10.1136/jmedgenet-2017-104877<br>DOI: 10.1002/ajmg.a.37501<br>W752 binds at the DNA fork. Mutation to C impacts DNA binding.
Q953K[b]	Q769	Undefi ned	C	Near C-end of motif VI Community connector (F and C)	DOI: 10.1590/1678-4685-GMB-2019-0085<br>DOI: 10.1136/jmedgenet-2017-104877<br>Q769 packs against the hinge loop between the RecA1 and RecA2 domains and forms a hydrogen bond with T740. Mutation to K likely impacts the hydrophobic packing of V773 and A742.
V957G[a]	V773	CS I	C	Near C-end of motif VI	DOI: 10.1590/1678-4685-GMB-2019-0085<br>DOI: 10.1136/jmedgenet-2017-104877<br>DOI: 10.1002/humu.21154

					V773 is near C-end of motif VI, packing with A742, A764 and W765 near the RecA1-RecA2 domain interface. V to G mutation disrupts hydrophobic interactions of these residues and affects helix/beta-sheet packing.
R975W[b]	R791	CS II	C	HD2-2 domain, DNA binding	DOI: 10.1590/1678-4685-GMB-2019-0085<br>DOI: 10.1136/jmedgenet-2017-104877<br>DOI: 10.3892/mmr.2017.6487<br>R791 is in HD2-2 domain and directly interacts with DNA. Mutation to W removes the favorable charged interaction and creates a clash with the DNA backbone.
L987P[b]	L803	COFS	C	HD2-2 domain Community connector (F, C and R)	DOI: 10.1590/1678-4685-GMB-2019-0085<br>DOI: 10.1136/jmedgenet-2017-104877<br>DOI: 10.1002/humu.21154<br>L803 is in the middle of helix HD2-2 domain. L to P mutation distorts the helix and disrupts HD1, CTD packing and F-C-R dynamic community interface.
W589del[b]	W369	CS I	F		DOI: 10.1590/1678-4685-GMB-2019-0085<br>DOI: 10.1136/jmedgenet-2017-104877<br>Deleting W369 shortens the sequence and prevents W368 from packing with ATP
L860del[b]	L672	CS II	C	Motif IV	DOI: 10.1590/1678-4685-GMB-2019-0085<br>DOI: 10.1136/jmedgenet-2017-104877<br>The deletion disrupts the ATPase hydrophobic core.
R467-R562del[b]	K253-G347	CS I	F	Deletes motif I	DOI: 10.1590/1678-4685-GMB-2019-0085<br>Deleting motif I prevents ATP binding and abolishes ATPase function.
Y510-R562del[b]	Y300-G347	CS II	F	Deletes motif I	DOI: 10.1590/1678-4685-GMB-2019-0085<br>Deleting motif I prevents ATP binding and abolishes ATPase function.
E608-Q723del[b]	D431-Q546	CS II	F, R	Deletes motif I, IIa, III and HD2-1	DOI: 10.1590/1678-4685-GMB-2019-0085<br>Deleting motif I, IIa, III and HD2-1 prevents ATP binding, abolishes ATPase activity and also prevents DNA binding.
F665-Q723del[a]	L488-Q546	CS II	F, R	Deletes motif III and the first half of HD1	DOI: 10.1590/1678-4685-GMB-2019-0085<br>DOI: 10.1002/humu.21154
V724-Q762del[b]	V547-M583	CS II	F, C, R	Deletes hinge between lobe1 and lobe2 and the second half of HD1	DOI: 10.1590/1678-4685-GMB-2019-0085<br>DOI: 10.1002/humu.21154<br>Deleting the hinge region prevents the opening/closing of the ATPase domains during the ATP hydrolysis cycle. Also impacts DNA association.
M752-Q762del[b]	K575-M583	COFS	C	Deletes hinge between RecA1 and RecA2	DOI: 10.1590/1678-4685-GMB-2019-0085<br>Deleting the hinge region prevents the opening/closing of the ATPase domains during the ATP hydrolysis cycle.
V763-Q794del[a]	V584-N615	CS II	C	Deletes part of HD2-1	DOI: 10.1590/1678-4685-GMB-2019-0085<br>DOI: 10.1002/humu.21154<br>Deleting HD2-1 prevents interactions with the upstream fork of the transcription bubble.

K538-T539delinsKNVF[b]	K328-T329	CS I	F	Disrupts motif I	DOI: 10.1177/0300060519877997 Deleting motif I prevents ATP binding and abolishes ATPase function.
P1042L[j]	Missing	CS I		N/A	DOI: 10.1590/1678-4685-GMB-2019-0085, DOI: 10.1136/jmedgenet-2017-104877 DOI: 10.1002/humu.21154
P1095R[j]	Missing	Undefined		N/A	DOI: 10.1136/jmedgenet-2017-104877 DOI: 10.1002/humu.21154
R1213G[j]	Missing	Undefined		N/A	DOI: 10.1136/jmedgenet-2017-104877
S1240-V1260delinsI(I)[bj]	Missing	CS III	N/A	N/A	DOI: 10.1590/1678-4685-GMB-2019-0085 DOI: 10.1002/humu.21154

[a]homozygote.
[b]heterozygote.
[c]Disease abbreviations: Cockayne syndrome (CS).
[d]C ID denotes dynamic community identifier. Communities are defined from dynamic network analysis as outlined under Methods.
[e]COFS –Cerebro-Oculo-Facio-Skeletal syndrome, a prenatal form of CS.
[f] Inferred function is the functional role of the mutation inferred from our structural and dynamic model.
[g]CS type I, a normal appearing newborn whose symptoms may not become apparent until after the first year.
[h]CS type II, obvious growth failure at birth along with little or no neurological development after birth.
[i]CS type III, rarer, essentially normal growth and mental development during the early years but interrupted by the late onset of the typical symptoms of CS.
[j]fall outside the extent of our structural model and could not be mapped.

Supplementary Table 3. Summary of Rad26 structural elements and original sources used for modeling

Protein	Size (aa)	Modeled Residues	Structures (PDB IDs) used for modeling
NTD	135	94-228	Residues 94-228 built *de novo*
NTD	68	229-297	Residues 229-297 revised and rebuilt based on existing 5VVR structure
NTD	18	298-315	Residues 298-315 taken from 5VVR
RecA1	242	316-558	Residues 316-558 taken from 5VVR
Linker[a]	86	559-577	Residues 559-577 revised and rebuilt based on existing 5VVR structure
RecA2	220	578-797	Residues 578-797 taken from 5VVR. Residues 632-644 built using modeler
RecA2	63	798-861	Residues 798-860 constructed by homology modeling using yeast *T. thermophilus* ISWI (PDB ID: 5JXR), *S. cerevisiae* SWI2/SNF2 (PDB ID: 5X0Y), and *M. thermophila* SNF2 (PDB ID: 5HZR) as templates
CTD	225	861-1085	Residues 861-1085 built *de novo*

[a]Linker between RecA1 and RecA2
