# Supplementary material for: Mechanism of Rad26-assisted rescue of stalled RNA polymerase II in transcription-coupled repair
Source: Nat Commun. 2021 Dec 1;12:7001. doi: 10.1038/s41467-021-27295-4 (PMC8636621; doi:10.1038/s41467-021-27295-4)
Supplement: Supplementary file 2 — Description of Additional Supplementary Files [file 41467_2021_27295_MOESM2_ESM.pdf]

Description of Additional Supplementary Files

File Name: Supplementary Movie 1
Description: **Pol II– Rad26 structural model, including the CTD and NTD domains**. Positions of Cockayne syndrome disease mutations are shown as spheres and colored by phenotype.

File Name: Supplementary Movie 2
Description: **Conformational changes in Rad26 in response to changed nucleotide state.** Rad26 is colored by domains. DNA and RNA are shown in blue and red. Cockayne syndrome disease mutations (red sphere) are mapped onto the Rad26 structure.

File Name: Supplementary Movie 3
Description: **Consensus communities identified from dynamic contact network analysis and mapped to the structure of the Pol II–Rad26 complex.** Dynamic communities are color-coded and labeled.
