# Supplementary material for: Mechanism of Rad26-assisted rescue of stalled RNA polymerase II in transcription-coupled repair
Source: Nat Commun. 2021 Dec 1;12:7001. doi: 10.1038/s41467-021-27295-4 (PMC8636621; doi:10.1038/s41467-021-27295-4)
Supplement: Supplementary file 6 — Reporting Summary [file 41467_2021_27295_MOESM6_ESM.pdf]

nature portfolio

Corresponding author(s): Ivaylo Ivanov
Dong Wang

Last updated by author(s): Nov 5, 2021

Reporting Summary

Nature Portfolio wishes to improve the reproducibility of the work that we publish. This form provides structure for consistency and transparency in reporting. For further information on Nature Portfolio policies, see our Editorial Policies and the Editorial Policy Checklist.

Statistics

For all statistical analyses, confirm that the following items are present in the figure legend, table legend, main text, or Methods section.

n/a	Confirmed	
☐	☒	The exact sample size (n) for each experimental group/condition, given as a discrete number and unit of measurement
☐	☒	A statement on whether measurements were taken from distinct samples or whether the same sample was measured repeatedly
☐	☒	The statistical test(s) used AND whether they are one- or two-sided<br>*Only common tests should be described solely by name; describe more complex techniques in the Methods section.*
☒	☐	A description of all covariates tested
☒	☐	A description of any assumptions or corrections, such as tests of normality and adjustment for multiple comparisons
☐	☒	A full description of the statistical parameters including central tendency (e.g. means) or other basic estimates (e.g. regression coefficient) AND variation (e.g. standard deviation) or associated estimates of uncertainty (e.g. confidence intervals)
☐	☒	For null hypothesis testing, the test statistic (e.g. F, t, r) with confidence intervals, effect sizes, degrees of freedom and P value noted<br>*Give P values as exact values whenever suitable.*
☒	☐	For Bayesian analysis, information on the choice of priors and Markov chain Monte Carlo settings
☒	☐	For hierarchical and complex designs, identification of the appropriate level for tests and full reporting of outcomes
☒	☐	Estimates of effect sizes (e.g. Cohen's d, Pearson's r), indicating how they were calculated

Our web collection on statistics for biologists contains articles on many of the points above.

Software and code

Policy information about availability of computer code

Data collection	AMBER 18 https://ambermd.org/
Data analysis	COOT 0.9.5 https://www2.mrc-lmb.cam.ac.uk/personal/pemsley/coot/<br>AmberTools 18 http://ambermd.org/AmberTools.php<br>MDTraj 1.9.5 https://www.mdtraj.org/1.9.5/index.html<br>NetworkX 2.2 https://networkx.github.io/<br>SOAN 1.0 https://github.com/tdodd3/SOAN<br>UCSF Chimera 1.12 https://www.cgl.ucsf.edu/chimera/<br>PHENIX 1.14 http://www.phenix-online.org/

For manuscripts utilizing custom algorithms or software that are central to the research but not yet described in published literature, software must be made available to editors and reviewers. We strongly encourage code deposition in a community repository (e.g. GitHub). See the Nature Portfolio guidelines for submitting code & software for further information.

Data

Policy information about availability of data

All manuscripts must include a data availability statement. This statement should provide the following information, where applicable:

- Accession codes, unique identifiers, or web links for publicly available datasets
- A description of any restrictions on data availability
- For clinical datasets or third party data, please ensure that the statement adheres to our policy

The data that support the findings of this study are available from the corresponding authors upon reasonable request. The models of Pol II-Rad26 in apo, ATP-

bound and ADP-bound states have been deposited in the ModelArchive database with DOI accession codes: 10.5452/ma-hxt70, 10.5452/ma-bd9wm and 10.5452/ma-51iiv, respectively. Source data are provided as a Source Data file.

Field-specific reporting

Please select the one below that is the best fit for your research. If you are not sure, read the appropriate sections before making your selection.

- [x] Life sciences
- [ ] Behavioural & social sciences
- [ ] Ecological, evolutionary & environmental sciences

For a reference copy of the document with all sections, see nature.com/documents/nr-reporting-summary-flat.pdf

Life sciences study design

All studies must disclose on these points even when the disclosure is negative.

Sample size	Snapshots from the MD trajectories were collected at intervals of 2.0 ps from a cumulative 33 microseconds of MD trajectory data. Sufficiently long intervals between collected frames were chosen to ensure selection of statistically uncorrelated conformations. 100,000 conformations from the MD trajectories of each functional state (apo, ATP-bound and ADP-bound) were used for clustering analysis to identify the dominant conformations, for dynamic network analysis with dCNA and for suboptimal paths analysis. The number of frames is sufficient to produce converged averages.<br>The biochemical assays on the CSB mutant variants (Fig. 6B) were repeated at least three times. Exact number of repeats for each mutant is included in the Source Data file. This is standard practice in the field and ensures at least 67% chance that the averages of the repeats is more accurate than a single measurement.
Data exclusions	No data were excluded from the analysis.
Replication	The biochemical assays in Fig 6B were repeated at least three times for each mutant. All attempts at replication were successful.
Randomization	Randomization is not relevant to this study as samples were not allocated to groups.
Blinding	Investigators were not blinded as this is not compatible with the methods used in the study. Blinding was not relevant as experimental procedures were standardized and all data points were included in the results.

Reporting for specific materials, systems and methods

We require information from authors about some types of materials, experimental systems and methods used in many studies. Here, indicate whether each material, system or method listed is relevant to your study. If you are not sure if a list item applies to your research, read the appropriate section before selecting a response.

Materials & experimental systems

n/a	Involved in the study	
x		Antibodies
x		Eukaryotic cell lines
x		Palaeontology and archaeology
x		Animals and other organisms
x		Human research participants
x		Clinical data
x		Dual use research of concern

Methods

n/a	Involved in the study	
x		ChIP-seq
x		Flow cytometry
x		MRI-based neuroimaging
